# Microglia-derived CCL2 has a prime role in neocortex neuroinflammation

**DOI:** 10.1186/s12987-022-00365-5

**Published:** 2022-08-30

**Authors:** Mariella Errede, Tiziana Annese, Valentina Petrosino, Giovanna Longo, Francesco Girolamo, Ignazio de Trizio, Antonio d’Amati, Antonio Uccelli, Nicole Kerlero de Rosbo, Daniela Virgintino

**Affiliations:** 1 Department of Basic Medical Sciences, Neuroscience, and Sensory Organs, University of Bari School of Medicine, Piazza Giulio Cesare, Policlinics, 70124 Bari, Italy; 2 Department of Medicine and Surgery, LUM University, Casamassima Bari, Italy; 3 Department of Neurosciences, Rehabilitation, Ophthalmology, Genetics, Maternal and Child Health, University of Genoa, Genoa, Italy; 4 Department of Emergency and Organ Transplantation, Pathology Section, University of Bari School of Medicine, Bari, Italy; 5 IRCCS Ospedale Policlinico San Martino, Genoa, Italy; 6 TomaLab, Institute of Nanotechnology, Consiglio Nazionale Delle Ricerche (CNR), Rome, Italy

**Keywords:** Experimental autoimmune encephalomyelitis, Neocortex, Microglia, CCL2, Mesenchymal stem cells

## Abstract

**Background:**

In myelin oligodendrocyte glycoprotein (MOG)-induced experimental autoimmune encephalomyelitis (EAE), several areas of demyelination are detectable in mouse cerebral cortex, where neuroinflammation events are associated with scarce inflammatory infiltrates and blood–brain barrier (BBB) impairment. In this condition, the administration of mesenchymal stem cells (MSCs) controls neuroinflammation, attenuating astrogliosis and promoting the acquisition of stem cell traits by astrocytes. To contribute to the understanding of the mechanisms involved in the pathogenesis of EAE in gray matter and in the reverting effects of MSC treatment, the neocortex of EAE-affected mice was investigated by analyzing the cellular source(s) of chemokine CCL2, a molecule involved in immune cell recruitment and BBB-microvessel leakage.

**Methods:**

The study was carried out by immunohistochemistry (IHC) and dual RNAscope IHC/in situ hybridization methods, using astrocyte, NG2-glia, macrophage/microglia, and microglia elective markers combined with CCL2.

**Results:**

The results showed that in EAE-affected mice, hypertrophic microglia are the primary source of CCL2, surround the cortex neurons and the damaged BBB microvessels. In EAE-affected mice treated with MSCs, microgliosis appeared diminished very soon (6 h) after treatment, an observation that was long-lasting (tested after 10 days). This was associated with a reduced CCL2 expression and with apparently preserved/restored BBB features. In conclusion, the hallmark of EAE in the mouse neocortex is a condition of microgliosis characterized by high levels of CCL2 expression.

**Conclusions:**

This finding supports relevant pathogenetic and clinical aspects of the human disease, while the demonstrated early control of neuroinflammation and BBB permeability exerted by treatment with MSCs may have important therapeutic implications.

**Supplementary Information:**

The online version contains supplementary material available at 10.1186/s12987-022-00365-5.

## Background

Experimental autoimmune encephalomyelitis (EAE), induced by myelin oligodendrocyte glycoprotein (MOG), is an animal model used widely for multiple sclerosis (MS), characterized by central nervous system (CNS) inflammation, demyelination, and axonal damage. EAE offers a good perspective of the human disease [1–3], which involves not only the white matter, as indeed the spinal cord is the main focus of immune attack in EAE with wide demyelination, but also the gray matter [4–13]. The inflammatory condition that spreads to the gray matter contributes to the clinical progression of the disease and is responsible for many of the observed neurological symptoms, including cognitive deficits, schizophrenia and mood disorders, the risk of seizures, and motor disabilities [14–17]. It is noteworthy that recent studies of cortical biopsies have shown that gray matter demyelination and the accompanying meningeal inflammation are not exclusive hallmarks of progressive disease, but can also be a major feature of even the earliest disease stages [18]. Among the chemokines, whose coding genes and pathways have been demonstrated to support the (etio)-pathogenesis of the inflammatory response [19, 20], the most extensively assessed is the chemokine (C–C motif) ligand 2 (CCL2), initially denominated monocyte chemoattractant protein-1 (MCP-1) [21]. CCL2 is known to participate in monocyte and T-cell recruitment, astrocyte activation, microglia migration/proliferation, and blood–brain barrier (BBB) dysfunction [22–27]. The CCL2 ligand and its receptor CCR2 have been amply investigated in post-mortem and biopsy spinal cord/brain tissue samples from MS patients, ex vivo samples from EAE-affected animals, and in studies in vitro on specific cell lineages [18]. Accordingly, astrocytes and endothelial cells are now widely accepted as the major resident cell sources of CCL2 in the CNS. The paucity of inflammatory infiltrates that characterizes the pathogenic profile of gray matter *vs.* white matter in EAE prompted us to investigate the response to inflammation of oligodendrocyte cell precursors, as well as the structural and functional status of BBB microvessels in the cerebral cortex of mice affected with MOG-induced EAE [10, 11, 28, 29]. In addition, we and others recently investigated the response of brain astrocytes in EAE, also monitoring the status of neuroinflammation and astrogliosis upon treatment with bone marrow-derived mesenchymal stem cells (MSCs) [30, 31]. MSC-based therapy has been demonstrated to improve the clinical course of autoimmune/neurodegenerative disorders, reducing neuroinflammation and promoting remyelination [32–36]. This possibly occurs through immunomodulatory and neuroprotective paracrine effects [32, 37, 38], or at a distance through MSC‐secreted factors and exosomes [32, 35, 39, 40], as well as through the activity of resident cell effectors, such as macroglia and microglia cells [30, 41]. In the present study, the neocortex of EAE-affected mice, featuring scarce monocyte/macrophage infiltrates, was used as a favorable setting for identifying the cell source(s) of CCL2 in the parenchyma as well as at the BBB-microvessel interface. A parallel, comparative study was made on EAE-affected mice treated with intravenous administration of MSCs and analyzed at different time intervals from disease onset. The study was performed by immunohistochemical (IHC) and dual IHC/RNAscope in situ hybridization (ISH) methods using macroglia and microglia elective markers combined with CCL2 immunolocalization and mRNA expression. BBB-microvessel junctional structure and barrier function were investigated by IHC analysis of tight junction (TJ) proteins, claudin-5 and occludin, together with FITC-dextran as a barrier permeability tracer. Overall, the study shows that in the neocortex of EAE-affected mice, the neuroinflammatory response consists of an impressive presence of CCL2, whose primary cell source has been identified as the activated, hypertrophic microglia. The microgliosis state, which also involves the barrier function of the cortex microvessels, appears to be effectively counteracted by MSC-based therapy, which leads to reduced microglia hypertrophy and CCL2 expression, an effect that lasted the duration of the experiment from the earliest (6 h) to the latest (10 days) time points examined after disease onset.

## Methods

### Ethics statement

All procedures involving animals were performed according to the guidelines of the Animal Ethical Committee and comply with the Decreto Legislativo 4 marzo 2014, n. 26, legislative transposition of Directive 2010/63/EU of the European Parliament and of the Council of 22 September 2010 on the protection of animals used for scientific purposes. All efforts were made to minimize the number of animals utilized. The research protocols were approved by the Ethics Committee for Animal Experimentation of the IRCCS Ospedale Policlinico San Martino and by the Italian Ministry of Health (Approval Nos. 398 and 444).

### EAE induction and treatment with MSCs

#### EAE induction

Female C579BL/6J mice were purchased from Harlan Laboratories (Udine, Italy) and housed in pathogen-free conditions with food and water ad libitum. Chronic EAE was induced at 6–8 weeks of age (average mouse weight 18.5 ± 1.5 g) by subcutaneous injection at two different sites in the right and left flanks and one site at the tail base with an emulsion (300 μl total) containing 200 μg myelin oligodendrocyte glycoprotein peptide spanning amino acids 35–55 (MOG_35–55_) (Espikem) in incomplete Freund’s adjuvant (Sigma-Aldrich) supplemented with 300 μg Mycobacterium tuberculosis (strain H37RA; Difco). Mice were injected in the tail vein with 400 ng pertussis toxin (Sigma-Aldrich) in 100 μl of phosphate buffer saline solution (PBS, pH 7.6), both immediately and 48 h after the immunization. Control, naïve C57BL/6 female mice were treated in the same way, except that the inoculum did not contain MOG_35–55_. The onset of EAE clinical signs occurred at 12 days post-immunization (dpi). The mice were weighed and scored daily for clinical manifestations of EAE on a clinical score (cs) scale of 0–5 [42].

### MSC culture and treatment

Bone marrow-derived MSCs were isolated from 6 to 8 week old female C579BL/6J mice (Harlan Laboratories, Udine, Italy), as previously described [37]. MSCs were expanded and maintained in culture in Mesencult basal medium supplemented with Mesenchymal Stem Cell Stimulatory Supplement (StemCell Technologies, Vancouver, BC, Canada) at 37 °C in a humidified 5% CO_2_ incubator. Mature MSCs were defined by a stable CD45^**−**^ CD34^**−**^ CD11b^**−**^ CD9^+^ Sca-1^+^ CD44^+^ phenotype [37]. MSCs were detached with trypsin, washed 3 times with PBS without Ca^2+^ and Mg^2+^ (Sigma-Aldrich), and counted. Mice induced for EAE were injected in the tail vein with 10^6^ MSCs/mouse diluted in 250 μl PBS (Sigma-Aldrich) at disease onset, i.e., at 12 dpi, or with an equal volume of PBS.

### Animal groups and study design

A total of 33 immunized mice were included in the following experimental groups: (a) EAE-affected mice (PBS-treated, no MSCs) (n = 16), sacrificed at 6 h (n = 10; cs 1.5–2.5), 24 h (n = 4; cs 1.5–3.5), and 10 days (n = 2; cs 2–2.5) after disease onset/PBS treatment; (b) EAE-affected mice treated with MSCs (EAE MSC-treated) (n = 14), sacrificed following the same timeline at 6 h (n = 8; cs 1–2), 24 h (n = 4; cs 1–3), and 10 days (n = 2; cs 2–2.25) after disease onset/MSC treatment; groups (a) and (b) included mice injected in the tail vein with the exogenous permeability marker FITC-dextran and sacrificed at 6 h and 24 h (n = 2 *per* group) (see below); (c) an additional group of EAE-affected mice (n = 3), injected with green fluorescent protein (GFP)-MSCs (see below) and sacrificed 24 h later; (d) naïve controls, sacrificed at time points corresponding to those of EAE-affected mice (n = 4, n = 3, n = 2).

### Biodistribution and fate of MSCs after administration

The MSC distribution and persistence in tissues was analyzed to investigate the therapeutic potential of MSC treatment [43]. Therefore, viable MSCs marked by GFP were searched for in both brain and lung tissues according to the following transfection and immunolocalization protocols.

### Transfection of MSCs and immunofluorescence confocal analysis

MSCs were transfected with green fluorescent protein (GFP) lentivirus transfecting GFP under the control of the phosphoglycerate kinase (PGK) promoter, as follows: MSCs at passage 3 were seeded at the concentration of 3 × 10^5^ cells in a T-75 cm^2^ flask grown at 37 °C, 5% CO_2_ for 24 h. They were then transfected with 9 × 10^7^ viral particles at a multiplicity of infection of 20. Seventy-two hours later, MSCs were tested by FACS analysis to assess the percentage of GFP-expressing cells and the mean fluorescence intensity, and injected in the tail vein as above. Immunofluorescence analysis of GFP-MSC was carried out by confocal microscopy on brain and lung sections. Briefly, the brain and lungs were collected from EAE-affected mice treated with GFP-MSC and subjected to histological and immunostaining procedures (see Immunohistochemistry paragraph). The GFP signal was directly detected, or else amplified by staining with a polyclonal rabbit (pRb) anti-GFP antibody (Ab) and revealed with the appropriate secondary Ab (see Table 1) [44]. The results showed that the exogenously administered GFP-MSC were ‘trapped’ in the lungs [45] (Additional file 1: Figure S1), while they were not detectable in the brain parenchyma, neither directly nor after immunostaining for GFP.

**Table 1:** List of primary and secondary antibodies applied to IHC and RNAscope IHC/ISH

Primary Abs	Host IgG	Concentration (µg/µl)	Supplier	Catalogue No
CCL2/MCP1	Mo IgG	15.5	Novus biological	NBP2-22,115
CD31	Rat IgG	0.6	Abcam	Ab7388
CD45	Rat IgG	5	Novus biological	NB110-93,609
Claudin-5	Mo IgG_1_	16.6	Thermo fisher	18–7364 4C3C2
Collagen type IV	Rb IgG	5.0	Acris	R1041
GFAP	Rat IgG	6.7	Invitrogen	13–0300
GFAP	Rb IgG	10	Immunostar	22,522
GFP	Rb IgG	5.0	Molecular probes	A6455
IBA1	Gt IgG	5	Abcam	ab5076
NG2	Rb IgG	5.5	Millipore	AB5320
Occludin	Rb IgG	5.0	Thermo fisher	71–1500
SALL1	Rb IgG	5	Abcam	Ab31526
TMEM119	Mo IgG	10	Abcam	ab209064

Secondary Abs and reagents﻿﻿		Concentration (µg/µl)﻿	Supplier﻿	Catalogue No﻿
Biotinylated horse anti-mouse IgG		3.75	Vector	BA-2000
Biotinylated goat anti-rabbit IgG		3.75	Vector	BA-1000
Biotinylated goat anti-rat IgG		3.75	Vector	BA-9400
Biotinylated horse anti-goat IgG		3.75	Vector	BA-9500
Streptavidin-Alexa 488		6.6	Invitrogen	S-11223
Streptavidin-Alexa 555		6.6	Invitrogen	S-21381
Goat anti-rabbit Alexa 568		6.6	Invitrogen	A11011
Goat anti-rabbit Alexa 488		6.6	Invitrogen	A11070
Goat anti-rat Alexa 555		2.5	Invitrogen	A21434

### Immunohistochemistry

EAE-affected mice treated or not with MSCs, plus naïve controls, were transcardially perfused with 2% paraformaldehyde (PFA) and 0.2% glutaraldehyde PBS solution, under deep anesthesia (ketamine/xylazine cocktail, 90 mg and 4.5 mg/kg, respectively) by intraperitoneal injection. Whole brains were removed, halved, and post-fixed by immersion in the same fixative for 4 h at 4 °C, then washed in PBS overnight at 4 °C. Using a vibrating microtome (Leica Microsystem), serial sagittal sections. (30–35 μm thick), evenly spaced at 200 µm intervals, were cut from each hemisphere as free-floating serial sections to be stored in 0.02% PFA in PBS at 4 °C in a multiwell archive. Single and double immunostaining for confocal microscopy was carried out with primary antibodies (Abs) against occludin, claudin-5, receptor-type tyrosine-protein phosphatase C (CD45), ionized calcium binding adapter molecule 1 (IBA1), C–C motif chemokine 2 (CCL2), collagen type IV (COL IV), CD31 (or platelet endothelial cell adhesion molecule, PECAM-1), glial fibrillary acidic protein (GFAP), neuron-glial antigen 2 (NG2), also known as chondroitin sulphate proteoglycan 4 (CSP4), transmembrane protein 119 (TMEM119), sal-like protein 1 (SALL1) (Table 1). After adhesion on polylysine slides (Menzel-Glaser, GmbH, Braunschweig, Germany) by drying for 10 min at room temperature (RT), the sections were subjected to the following protocol: rehydration with PBS for 5 min at RT; either protease pre-treatment 0.1 mg/ml in PBS for 2 min at 37 °C (Proteinase K, Code No. 03115879001; Roche, Indianapolis, USA) or heat induced epitope retrieval (HIER) by microwave pre-treatment in 0.01 M citrate buffer (pH 6.0) for 15 min at 750 W (when required); incubation with 0.5% Triton X-100 (Merck) in PBS for 30 min at RT and with Vector M.O.M™ Immunodetection Kit (diluted 35 ml/ml PBS; Vector) 1 h at RT and/or Protein Block Serum Free (Dako) for 15 min at RT; incubation with single or combined primary Abs, diluted in blocking buffer (BB; PBS, 1% bovine serum albumin, 2% FCS; Dako) overnight (ON) at 4 °C, revealed by appropriate fluorophore-conjugated secondary Abs or biotinylated secondary Abs followed by fluorophore-conjugated streptavidin (Table 1), diluted in BB for 45 min at RT. After immunolabeling, the sections were fixed in 4% PFA for 10 min and counterstained with TO-PRO3 diluted 1:10 K in PBS (633; Invitrogen). After each incubation step, the sections were washed 3 times for 5 min with PBS. Finally, the sections were coverslipped with Vectashield (Vector) and sealed with nail varnish. Negative controls were prepared by omitting the primary antibodies and mismatching the secondary antibodies. The sections were examined under the Leica TCS SP5 confocal laser-scanning microscope (Leica Microsystems, Mannheim, Germany) using a sequential scanning procedure. Confocal images were taken at 250–500 nm intervals through the z-axis of the sections with 40 × and 63 × oil lenses. Z-stacks of serial optical planes (projection images) and single optical planes were analyzed with Leica confocal software (Multicolor Package; Leica Microsystems).

### Microvessel permeability assay

A heparin solution (100 Units/kg) containing Fluorescein isothiocyanate–dextran 70-kDa (5 mg/ml; FITC-dextran 70, Sigma-Aldrich), as a fluorescent probe to evaluate BBB-endothelial cell permeability, was injected into the tail vein of naïve, EAE-affected, and EAE-affected MSC-treated mice. Four minutes after the end of the injection, the mice were anesthetized, sacrificed by CO_2_ inhalation, and the brains were collected and fixed by immersion in the fixative solution (2% PFA plus 0.2% glutaraldehyde in PBS pH 7.4) ON at 4 °C. The tissues were then washed in PBS for 6 h at 4 °C and stored in PFA 0.02% at 4 °C. Sections (30–35 μm-thick) were immunolabeled with a pRb Ab anti-GFAP, revealed by the appropriate secondary Ab (Table 1), and counterstained with TO-PRO3, applying the same IHC method and confocal laser microscopy setting described above.

### Dual RNAscope-immunohistochemistry/in situ hybridization (IHC/ISH)

Brain sections from naïve, EAE-affected, and EAE MSC-treated mice were collected from the multiwell archive of sections (see Immunohistochemistry paragraph) and processed with ‘Dual IHC/ISH RNAscope Technology’ (Advanced Cell Diagnostic, ACD, Inc.; Hayward, CA, USA). We applied the method described for vibratome-cut brain sections. (46), adapted to detect first the GFAP protein and then *Ccl2* mRNA. After adhesion on polylysine slides by drying for 10 min at RT the sections were immunostained, according to the described IHC method, with a pRb Ab anti-GFAP (Table 1), ON at 4 °C, and revealed with Alexa fluor 488-conjugated secondary antibody for 45 min at RT. Then, the same slides were processed for RNA ISH using the RNAscope^®^ 2.5 HD Reagent Kit-RED [ACD, 322360]. Sections were (1) incubated in Pretreatment 1 (H_2_O_2_) for 60 min at RT; (2) rinsed in PBS (4 × 1 min); (3) dried for 1 h at RT; (4) incubated in Pretreatment 2 target retrieval reagent (ACD, 322330) for 7 min at 99–104 °C; (5) washed in H_2_O (2 × 1 min); (6) dried for 10 min at RT; (7) dipped in 100% ethanol and air-dried; (8) demarcated by a hydrophobic fence using ImmEdge Hydrophobic Barrier Pen (ACD, 310018); (9) incubated in Pretreatment 3 (protease, pretreatment kit; ACD, 322330) for 15 min at 40 °C in the EZ Hybridization oven (ACD, 310012) in a humidity control tray/slide rack (ACD, 310014); (10) washed (4 × 1 min) in H_2_O for 1 min; (11) incubated with mouse probe *Ccl2* (4 drops/section; ACD, 311791) for 2 h at 40 °C; (12) washed (2 × 1 min) in 1 × wash buffer (ACD, 310,091). Amplification (Amp) and detection were performed using the RNAscope 2.5 HD Reagent Kit-RED (ACD, 322350) in the humidity control tray/slide rack according to the following incubation steps: Amp1 for 30 min at 40 °C, Amp2 for 15 min at 40 °C, Amp3 for 30 min at 40 °C, Amp4 for 15 min at 40 °C, Amp5 for 30 min at RT, Amp6 for 15 min at RT. After each incubation step, the sections were rinsed in wash buffer (2 × 2 min). The ISH signal was detected by incubating the slides in a mixture of Fast-Red-A and Fast-Red-B solutions at a 1:60 ratio for 10 min at RT. The slides were washed in H_2_O (2 × 2 min), counterstained with Sytox Green (diluted 1:5 k in PBS for 10 min; Thermo Fisher Scientific), and finally coverslipped with ProLong Diamond Antifade Mountant (Thermo Fischer Scientific, P36961). The sections were examined under a Leica TCS SP5 confocal laser scanning microscope (Leica Microsystems) using a sequential scanning procedure to detect the fluorescence signal of both Alexa 488 and Fast-Red.

### Morphometric analyses

#### Morphometric analysis of CCL2-stained microglia

Morphometric analysis was performed on CCL2-stained brain sections from both EAE-affected (n = 4, 3 sections each) and EAE MSC-treated (n = 4, 3 sections each) mice at 24 h from the disease onset. Two independent observers (GL and FG), blinded for group allocation, assessed randomly selected fields at 63 × magnification, using computer-aided morphometry and ImageJ software (NIH, Bethesda, USA). The mean area encompassed by microglial cells was measured by FracLac plugin (http://rsb.info.nih.gov/ij/plugins/fraclac/FLHelp/Introduction.htm.1999–2013) as the ‘convex hull area’, the latter determined from the polygon created by straight lines connecting the most distal points of the cell processes. The ‘circularity’ (ratio between the area of the cell to the area of a circle having the same perimeter) and ‘number of endpoints’ (number of process terminals/thorns *per* cell) were evaluated on single binary CCL2-expressing microglia outlines using the FracLac plugin and the Analyze Skeleton (2D/3D) plugin (Arganda-Carreras, Fernández-González, Muñoz-Barrutia, & Ortiz-De-Solorzano, 2010), respectively. Corrected total immunofluorescence density of CCL2 was also calculated as integrated density − [image area × mean fluorescence of background reading], expressed as a arbitrary unit (AU) (https://theolb.readthedocs.io/en/latest/imaging/measuring-cell-fluorescence-using-imagej.html).

### Morphometric analysis by SALL1/IBA1- and GFAP/Ccl2-staining

Morphometric analysis was performed on brain sections from naïve (n = 3; 3 sections each), EAE-affected (n = 4; 3 sections each), and EAE MSC-treated (n = 4; 3 sections each) mice, at 24 h from the disease onset. Cell counting on SALL1/IBA1- and GFAP/Ccl2-stained sections was performed by two independent observers (GL and TA), using the Aperio ImageScope (Leica Biosystems) and calculating the number of SALL1^+^/IBA1¯, SALL1^+^/IBA1^+^, SALL1¯/IBA1^+^ GFAP¯/*Ccl2*^+^, GFAP^+^/*Ccl2*^+^, GFAP^+^/*Ccl2*¯ on the total cells counted on 3 randomly selected fields *per* section, acquired at 40 × magnification. Data were reported as percentage values. Measurement of SALL1 and *Ccl2* fluorescence intensity was performed on 3 randomly selected fields *per* section, acquired at 40 × magnification using Cell^F (Olympus Italia, Rozzano, Italy).

### Statistical analysis

All quantitative data were expressed as mean value ± SD. The morphometric analyses of CCL2-stained microglia were analyzed using unpaired t-test. The percentage of cells single or double-positive for IBA/SALL1 or GFAP/*Ccl2* was statistically analyzed using, regular two-way ANOVA, while the fluorescence intensity of cells positive for SALL1 or *Ccl2* was statistically analyzed using one-way ANOVA. For both, Bonferroni post-test with swap direction was applied to correct multiple comparisons (GraphPad Prism, GraphPad Software, Inc.). An alpha of 0.05 was set as the cut-off for significance.

## Results

### Analysis of neocortex from naïve, EAE, and EAE MSC-treated mice by IHC for elective cell markers and CCL2

**Fig. 1:**
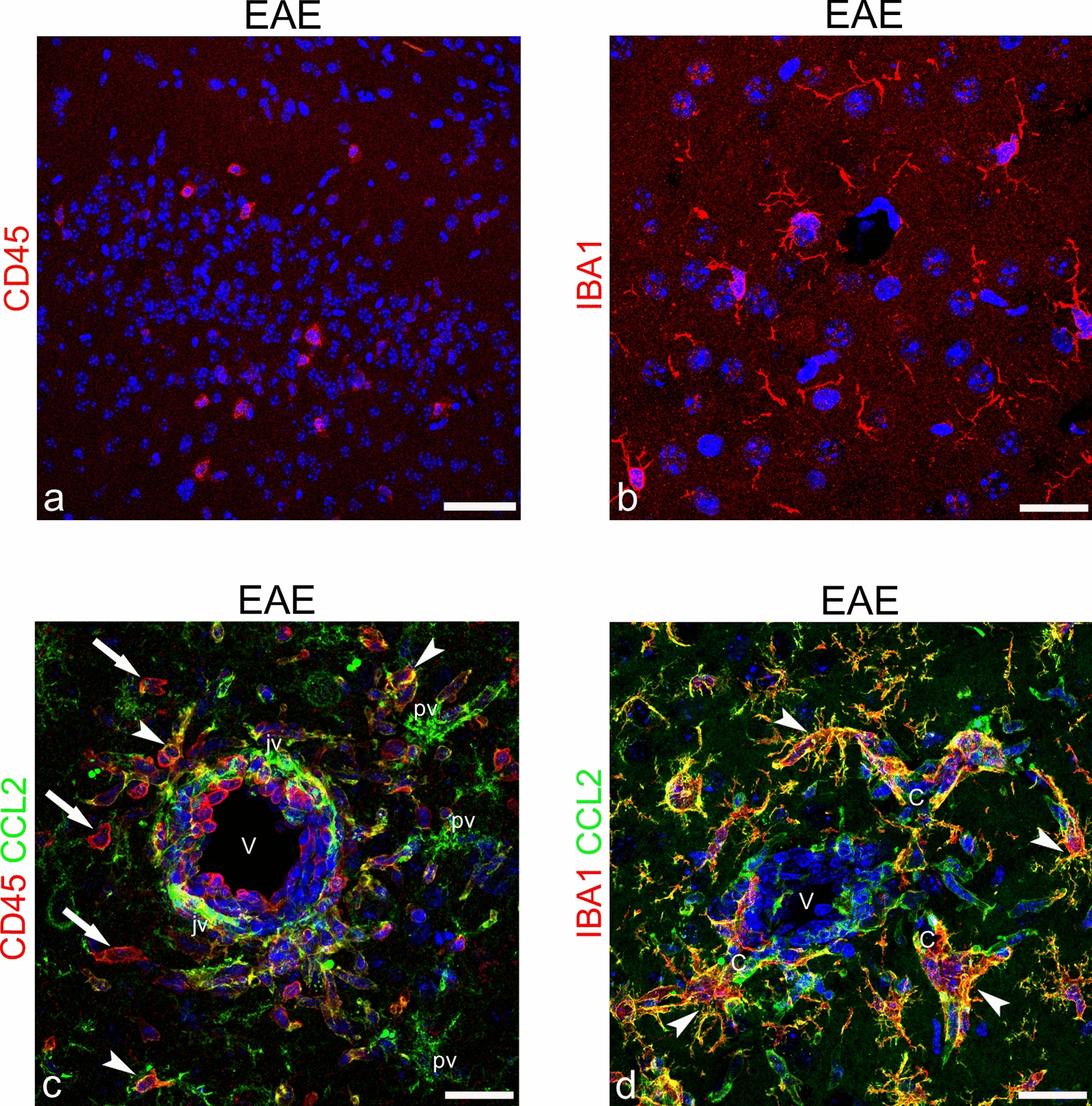
Representative images of neocortex sections from EAE-affected mice, sacrificed at 24 h from disease onset and immunostained for CD45 (a; cs 3.5), IBA1 (b; cs 3.5), CD45/CCL2 (c; cs 3.5), and IBA1/CCL2 (d; cs 3.5). **a** Low magnification of neocortex layers I-II shows a few, amoeboid, CD45^high^ monocyte/macrophage cells. **b** IBA1^+^ ramified, microglia-like cells are recognizable in the neocortex. **c** A venule (V) in the subcortical white matter surrounded by amoeboid, CD45^high^/CCL2^−^ infiltrating monocytes/macrophages (*arrows*), CCL2 colocalization with CD45 on perivascular cells (*arrowheads*), and CD45^−^/CCL2^+^ perivascular (ramified; pv) and juxtavascular (elongated; jv) cells. **d** In the same subcortical area, a postcapillary venule (V) and its closest capillary tracts (C) are surrounded by IBA1^+^/CCL2^+^ ramified cells (*arrowheads*). TOPRO-3 nuclear counterstaining. Scale bars: a 50 µm; b–d 25 µm

As a preliminary approach, the general grade of inflammation in the neocortex of EAE-affected mice was investigated in the experimental group sacrificed 24 h after disease onset. Single and double immunostainings were carried out for macrophage/microglia markers, CD45 and IBA1, and with antibody against CCL2 (Fig. 1). In the neocortex of EAE-affected mice, the scarce cellular infiltrate observed was mostly composed of amoeboid, CD45^high^ monocyte/macrophage cells scattered through the cortex layers, while ramified, CD45^low^ activated microglial cells were barely detectable [47, 48] (Fig. 1a). Microglia-like cells were better shown by IBA1 immunostaining that revealed numerous ramified cells throughout the cortex layers (Fig. 1b). In the subcortical white matter, CD45/CCL2 double immunostaining showed an infiltrate of amoeboid, CD45^high^/CCL2^−^ monocytes/macrophages, together with perivascular and juxtavascular ramified, CD45^−^/CCL2^+^ microglia-like cells (Fig. 1c); at the border with the deepest cortex layer, a large population of IBA1^+^/CCL2^+^ parenchymal and perivascular/juxtavascular microglia-like cells was predominant in the capillary and postcapillary venule vascular fields (Fig. 1d). It should be born in mind that *Iba1* is known to be expressed by brain-invading monocytes but only after these monocytes have entered the brain parenchyma and undergone maturation [49].

**Fig. 2:**
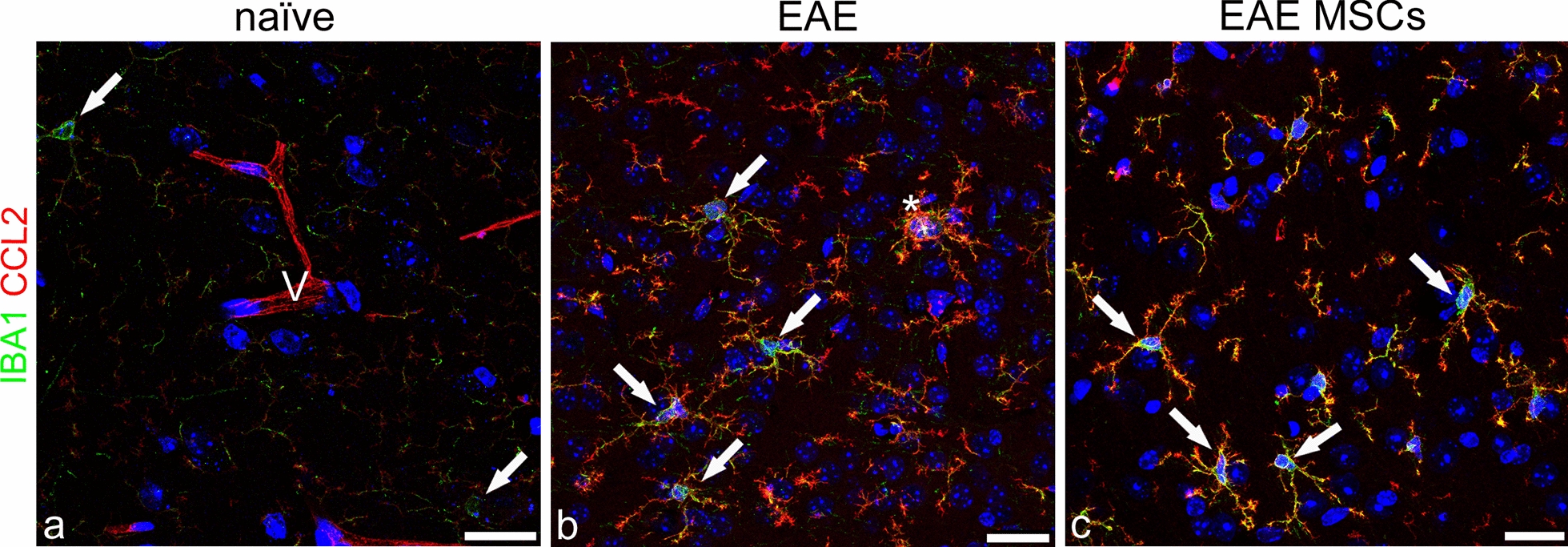
Representative images of neocortex sections from naïve (a), EAE-affected (b; cs 2.0) and EAE-affected MSC-treated (c; cs 1.5) mice, sacrificed 24 h after MSC treatment, double immunostained for IBA1 and CCL2. **a** Microglia-like cells are faintly stained by IBA1 (*arrows*) and a CCL2-stained microvessel is recognizable (V). **b** The two markers are extensively colocalized on microglia-like cells in the EAE neocortex, defining hypertrophic cells characterized by ‘thorny’ processes and a bushy aspect (*arrows*); IBA1 prevails on the cell body and is fully colocalized with CCL2 on cell processes; note aspects of cell fusion (*asterisk*). **c** In the cortex of EAE-affected MSC-treated mice, IBA1/CCL2-stained microglia-like cells show a simplified morphology and smaller size but the same distribution of the two markers (*arrows*). TOPRO-3 nuclear counterstaining. Scale bars: a–c 25 μm

According to these preliminary results, the analysis of microglia activation in the neocortex was carried out by IBA1/CCL2 double staining in EAE-affected MSC-treated and untreated mice, as well as in naïve controls (Fig. 2). In the latter, faint staining for IBA1 was characteristic of the surveillant microglia, whereas any CCL2 immunosignal was detected throughout the parenchyma, except for the wall of neocortex microvessels, whose endothelial cells showed a constitutive and ubiquitous expression of the chemokine (Fig. 2a). In EAE-affected mice, a large population of IBA1^+^/CCL2^+^ cells was observed, with hypertrophic bodies and multiple, short, thorny processes (Fig. 2b), whereas in EAE MSC-treated mice, IBA1^+^/CCL2^+^ cells showed an attenuated reactive morphology (Fig. 2c; see also the Morphometric analyses paragraph). It has been emphasized that the morphological and phenotypic differentiation between parenchymal or perivascular macrophages and the ‘real’ resident microglia is still a major challenge [50–52]. However, when observed in ultrastructural detail, activated microglial cells, unlike bone marrow cells and tissue macrophages, show process surfaces with spikes [53, 54], a feature that is well recognizable by confocal microscopy and that supports the recognition of these cells as microglial cells (Fig. 2b, c).

**Fig. 3:**
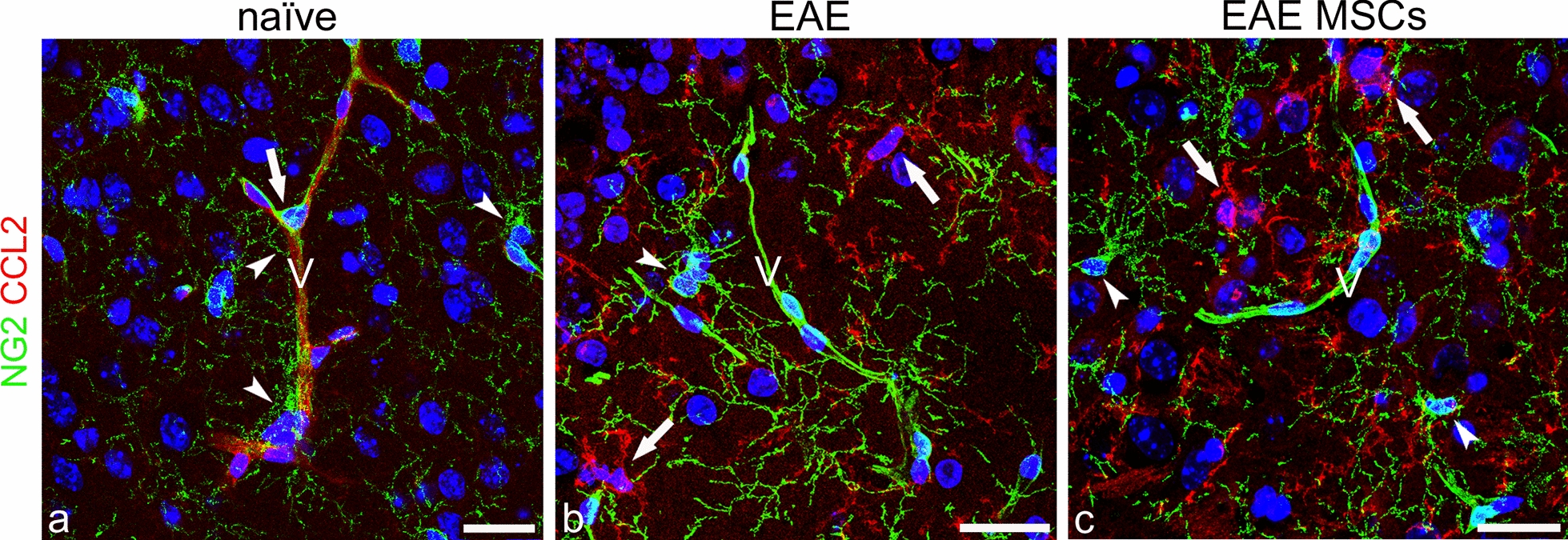
Representative images of neocortex sections from naïve (a), EAE-affected (b; cs 3.5), and EAE-affected MSC-treated (c; cs 2.25) mice, sacrificed 24 h after MSC treatment, and double immunostained for NG2 and CCL2. **a** NG2-glia is recognizable (*arrowheads*) also associated to the microvessel wall (V); note an NG2-reactive pericyte (*arrow*) and the CCL2 staining of the microvessel (V). **b**, **c** NG2 is not colocalized with CCL2 on the activated NG2-glia (*arrowheads*), characterized by increased branching and NG2 upregulation, whereas the chemokine appears restricted to NG2^−^ macrophage/microglia-like ramified cells (*arrows*). TOPRO-3 nuclear counterstaining. Scale bars: a–c 25 μm

To investigate the expression of CCL2 by astrocytes and NG2-glia, double immunostaining was performed for the specific cell markers GFAP and NG2, respectively. NG2 is a major CNS chondroitin sulfate proteoglycan, which is expressed by oligodendrocyte precursor cells (OPCs) and NG2-glia (adult OPCs), as well as by immature/re-activated pericytes [55]. It is now known that NG2-glia may respond to CNS injury with a state of activation/dedifferentiation characterized by the overexpression of NG2 [10, 29, 56]. The double immunolabeling experiments showed that CCL2 was never colocalized with NG2 on NG2-glial cells (Fig. 3a–c). During EAE, NG2-glia demonstrated an activated phenotype characterized by hypertrophy, an increased number of extensive processes, and strong NG2 staining (Fig. 3b, c). Moreover, in the neocortex of EAE-affected mice, a recognizable NG2^−^/CCL2^+^ population of ramified macrophage/microglia-like cells was also detected, tightly intermingled with NG2^+^ cell bodies and processes (Fig. 3b, c).

**Fig. 4:**
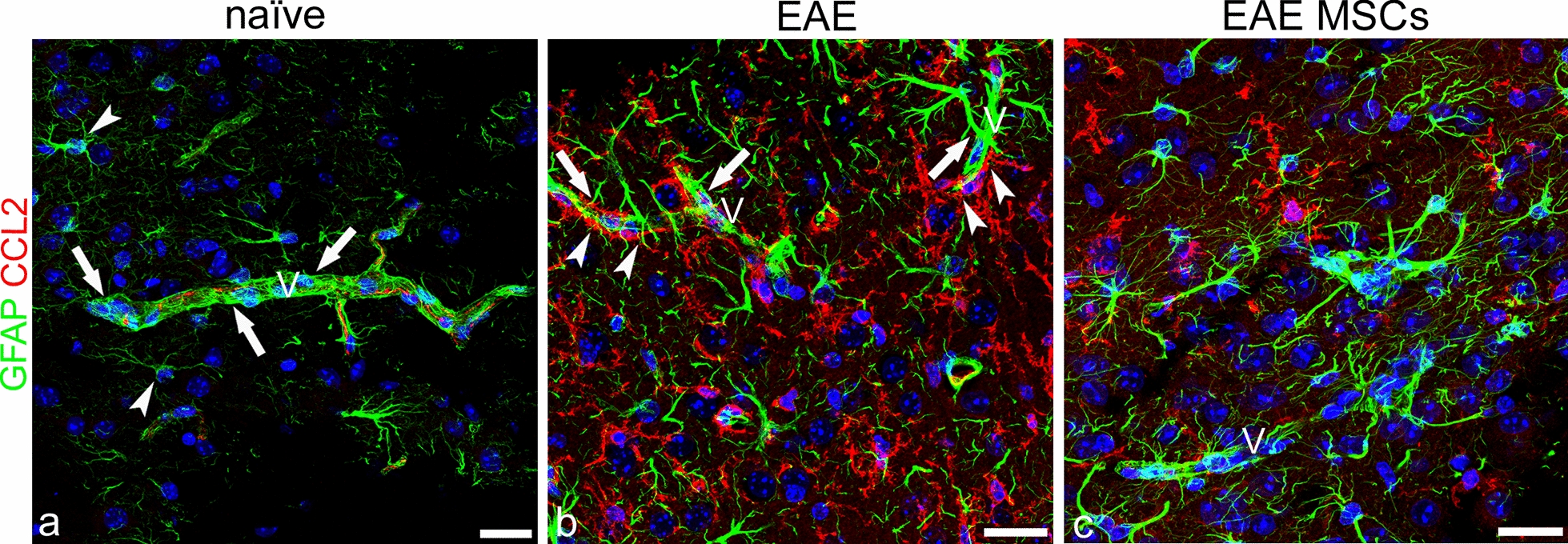
Representative images of neocortex sections from naïve (a), EAE-affected (b; cs 2.0) and EAE-affected MSC-treated (c; cs 1.5) mice, sacrificed 24 h after MSC treatment, and double immunostained for GFAP and CCL2. **a** GFAP-reactive astrocytes are recognizable, scattered in the parenchyma (*arrowheads*) and concentrated perivascularly (*arrows*); note the CCL2 staining on the microvessel wall (V). **b** In EAE-affected mice, the neocortex is characterized by diffuse astrogliosis with hypertrophic astrocytes that express high levels of GFAP; the microvessels (V) are ensheathed by GFAP^+^ perivascular endfeet (*arrows*) together with CCL2^+^ microglia-like processes (*arrowheads*). **c** The condition shows recovery in EAE-affected MSC-treated mice, where GFAP^+^ perivascular astrocytes once again prevail on the vessel wall (V). TOPRO-3 nuclear counterstaining. Scale bars: a–c 25 µm

GFAP is the classical marker of astrocytes, and is increased in the astrogliosis state that characterizes the response of astrocytes to neurologic insults [57]. The double GFAP/CCL2 immunostaining did not detect the chemokine in astrocytes either in naïve or in EAE-affected mice (Fig. 4a–c). Interestingly, a detectable and continuous CCL2 staining revealed neocortex microvessels in naïve mice (Fig. 4a; see also Figs. 2a and 3a). This aspect was further investigated in all the experimental groups by double staining with CCL2 and collagen type IV (COL IV), a main molecular component of the vessel basal lamina, or CD31 as an endothelial cell-specific marker. The analysis suggested the existence, in the neocortex of naïve mice, of a constitutive endothelial expression of CCL2 (Additional file 2: Figure S2a, d, g; Additional file 3: Figure S3a) that was considerably reduced in both EAE-affected (Additional file 2: Figure S2b, e, h; Additional file 3: Figure S3b) and EAE-affected MSC-treated (Additional file 2: Figure S2c, f, i; Additional file 3: Figure S3c) mice. As expected, on GFAP/CCL2 double stained sections from the neocortex of EAE-affected mice, astrocytes showed morphological features of astrogliosis, with cell hypertrophy and GFAP overexpression (Fig. 4b). Hypertrophic astrocytes displayed stout processes and shared their perivascular location with an overwhelming presence of CCL2^+^ cell processes, that were never colocalized with GFAP (Fig. 4b). The level of astrogliosis was decreased in EAE-affected MSC-treated mice and finer astrocyte processes and endfeet were again prevalent on the microvessel wall (Fig. 4c). In the neocortex of these treated mice, the perineuronal CCL2^+^ processes were also reduced compared with EAE-affected untreated mice (Fig. 4b, c; see also the Morphometric analyses paragraph).

### Confirmation of activated microglia as source of CCL2

**Fig. 5:**
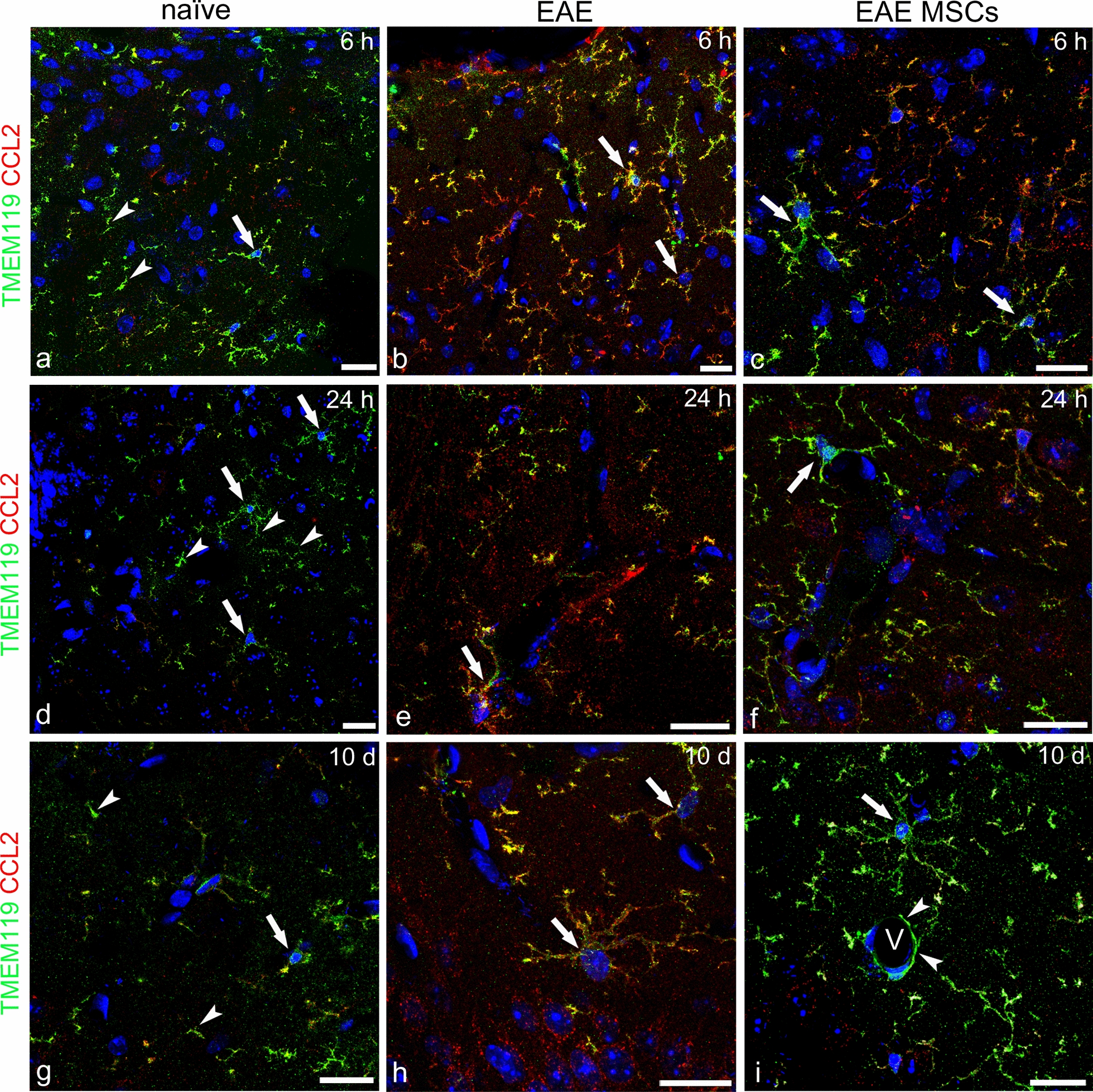
Representative images of neocortex sections from naïve (a, d, g), EAE-affected (b, e, h; cs 1.5, 2.0, 2.5, respectively), and EAE-affected MSC-treated (c, f, i; cs 1.0, 1.5, 2.0, respectively) mice, sacrificed at 6 h (**a–c**), 24 h (**d**–**f**), and 10 days (**g**–**i**) after MSC treatment, double immunostained for TMEM119 and CCL2. **a**, **d**, **g** In naïve mice, the typical delicate morphology of surveillant microglia is revealed by TMEM119 staining of cell bodies (*arrows*) and processes (*arrowheads*). **b**, **e**, **h** In EAE mice, TMEM119 is extensively colocalized with CCL2 on hypertrophic microglial cells, with the chemokine staining largely prevailing on cell bodies and processes (yellowish/reddish fluorescence; *arrows*). **c**, **f**, **i** Microglia hypertrophy and CCL2 staining are reduced on microglia of EAE-affected MSC-treated mice, with reduced cell points of CCL2/TMEM119 colocalization (yellowish fluorescence) and a prevailing TMEM119 staining (green fluorescence; *arrows*); note in (i) TMEM119-positive microglia processes that surround the wall of a cortex microvessel (V, *arrowheads*). TOPRO-3 nuclear counterstaining. Scale bars: a–i 20 µm

Considering the morphological features of the CCL2^+^ ramified/thorny cells observed in the neocortex of EAE-affected mice and the fact that CCL2 was never colocalized with NG2-glia or GFAP^+^ astrocytes, we used microglia-specific markers such as TMEM119 and SALL1 [58–60] to evidence and monitor the ‘real’ resident microglia. In the neocortex of naïve mice, CCL2/TMEM119 double staining showed the finely ramified profile of CCL2^−^/TMEM119^+^ surveillant microglia (Fig. 5a, d, g), whereas in EAE-affected mice, at all the time points considered, the two markers were colocalized on activated microglia, which were characterized by the presence of prominent, short cellular processes and the strong expression of CCL2 (Fig. 5b, e, h). As early as at 6 h after intravenous injection of EAE-affected mice with MSCs, microgliosis was reduced and the intensity of CCL2 staining on the microglia was faint (Fig. 5c, f, i). In some of the microglial cells, CCL2 expression was strongly downregulated, albeit not completely abolished, and TMEM119 staining prevailed, thus confirming that treatment with MSCs attenuates the phenotype of activated microglia (Fig. 5c, f, i). In short, the double CCL2/TMEM119 immunostaining definitively confirmed that the ramified population of cells that appeared activated and scattered throughout the neocortex layers of EAE-affected mice, as well as those concentrated in the perivascular area, were ‘real’ resident microglia.

**Fig. 6:**
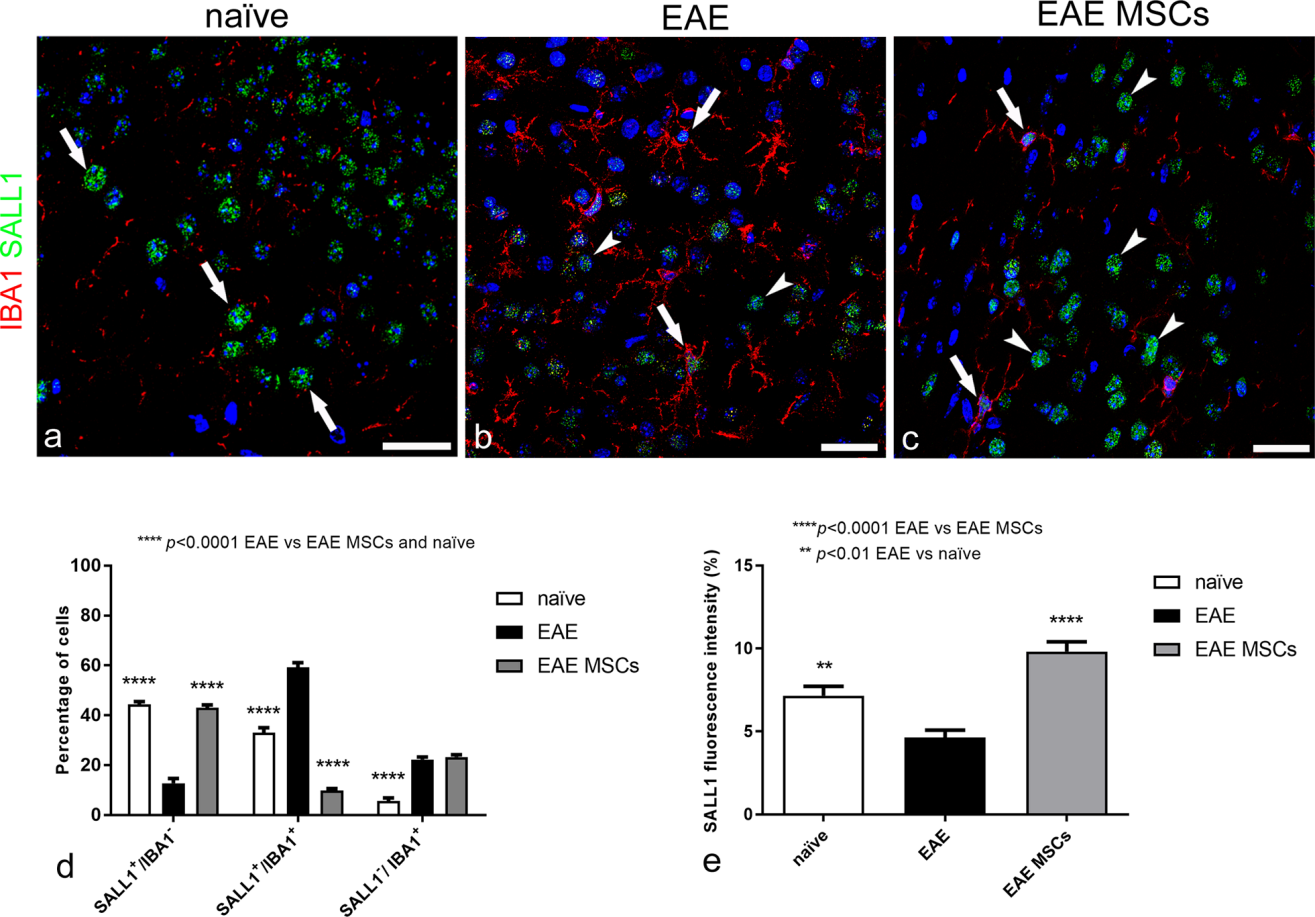
Representative images of neocortex sections from naïve (a), EAE-affected (b; cs 2.0) and EAE-affected MSC-treated (c; cs 1.5) mice, sacrificed 24 h after MSC treatment, double immunostained for IBA1 and SALL1; morphometric analyses of IBA1/SALL1 cell populations and SALL1 fluorescence intensity (d, e). **a** SALL1 specifically marks the nucleus of surveillant microglia, which express low levels of IBA1 (SALL1^+^/IBA1^−^ cell population, *arrows*). **b** SALL1^+^/IBA1^+^ (*arrows*) and SALL1^−^/IBA1^+^ (*arrowheads*) cell populations are both recognizable. **c** Surveillant SALL1^+^/IBA1^−^ microglia (*arrows*) prevail on SALL1^+^/IBA1^+^ reactive microglia (*arrowhead*). **d** In EAE-affected mice, the percentage of surveillant SALL1^+^/IBA1^−^ microglia strongly declines in favor of a significant increase in reactive SALL1^+^/IBA1^+^ microglia and SALL1^−^/IBA1^+^ monocytes/macrophages. In EAE-affected MSC-treated mice, the percentage of SALL1^+^/IBA1^−^ is similar to the level observed in naïve mice, whereas the increase in SALL1^+^/IBA1^+^ microglia observed in EAE-affected mice appears reverted by the treatment with MSCs; note the equal elevated percentage of SALL1^−^/IBA1^+^ monocytes/macrophages in both treated and not treated EAE-affected mice. **e** Fluorescence intensity for SALL1 is significantly higher in naïve mice microglia compared to EAE-affected mice; indeed, the highest levels are seen in EAE-affected MSC-treated mice. Data are reported as means ± SD (n = 3, n = 4, n = 4; 3 sections each), and the Bonferroni post-test was used to compare all groups after two-way ANOVA (d) or one-way ANOVA (e). Clinical score of EAE-affected mice (mean cs 2.1) and MSC-treated mice (mean cs 2.0). The TOPRO-3 nuclear counterstaining. TOPRO-3 nuclear counterstaining. Scale bars: a–c 25 µm

Microglial cells were also identified by the microglial cell-specific marker SALL1 (Fig. 6), a zinc finger transcriptional repressor that belongs to the SALL-like family of transcription factors and is involved in cerebral cortex neurogenesis. SALL1 is specifically expressed in human and mouse microglia, as compared with other macrophage populations, and its absence in microglia cells has been correlated with the appearance of a proinflammatory and phagocytic phenotype [59, 61]. In addition, taking advantage of the divergent locations of SALL1 in the nucleus *vs*. IBA1 in the cytoplasm, we were able to carry out a comparative, morphometric analysis of the diverse microglia and monocyte/macrophage populations (Fig. 6a–d), namely SALL1^+^/IBA1^−^, SALL1^+^/IBA1^+^, and SALL1^−^/IBA1^+^, corresponding to resting microglia, activated microglia, and monocytes/macrophages, respectively (Fig. 6d). Morphometric analyses revealed that, as expected, SALL1^+^/IBA1^−^ surveillant microglia were the main population in the cortex of naïve control mice (Fig. 6a, d), a feature mirrored in EAE-affected MSC-treated mice (Fig. 6c, d), which contrasted strikingly with the small percentage of resting microglial cells detected in untreated EAE-affected mice (Fig. 6b, d). In the latter, SALL1^+^/IBA1^+^ activated microglia prevailed, whereas this population strongly decreased after MSC treatment, even below the levels observed in naïve mice (Fig. 6d). The SALL1^−^/IBA1^+^ monocyte/macrophage population, which was elevated in EAE, was not affected by MSC treatment (Fig. 6d). Fluorescence intensity values detected for SALL1 indicated a reduced expression in EAE-affected mice, whereas SALL1 appeared increased in EAE-affected MSC-treated mice, showing even higher levels than in naïve mice (Fig. 6e).

### Application of dual IHC/ISH RNAscope technology to GFAP protein/*Ccl2* mRNA analysis

**Fig. 7:**
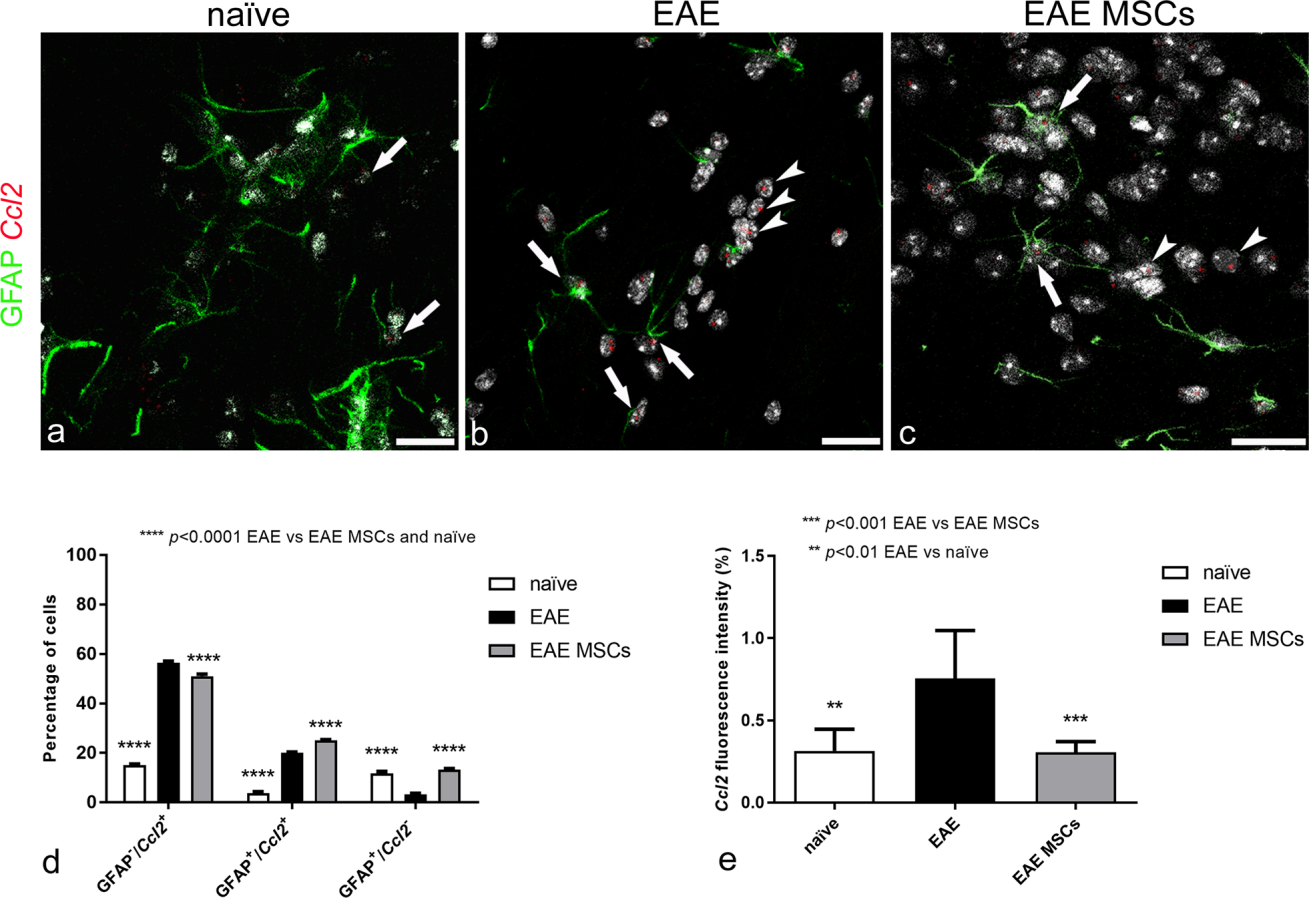
Representative images of neocortex sections from naïve (a), EAE-affected (b; cs 2.0) and EAE-affected MSC-treated (c; cs 1.0) mice, sacrificed 24 h after MSC treatment, and processed according to the dual RNAscope IHC/ISH method applied to concomitant detection of GFAP protein and *Ccl2* mRNA; morphometric analyses of GFAP/*Ccl2* cell populations and *Ccl2* fluorescence intensity (d, e). **a** Few GFAP^+^ astrocytes show a barely detectable expression of *Ccl2* (*arrows*) in naïve mice. **b** In EAE-affected mice, GFAP^+^ astrocytes express high levels of *Ccl2* (*arrows*), the *Ccl2* probes also marking a large GFAP^−^ cell population (*arrowheads*). **c** A few *Ccl2* expressing cells, both GFAP^+^ (*arrows*) and GFAP^−^ (*arrowheads*), are recognizable in MSC-treated mice. **d** Morphometric quantitation of GFAP^−^/*Ccl2*^+^ cells shows a significant increase in this cell population in EAE-affected mice compared with naïve controls; this increase is also detected, although to a significantly lesser extent, in EAE-affected MSC-treated mice; in both treated and not treated EAE-affected mice, GFAP^+^/*Ccl2*^+^ astrocytes are significantly more numerous compared to naïve mice, whereas the neocortex of EAE-affected MSC-treated mice is characterized by the prevalence of the GFAP^+^/*Ccl2*^−^ astrocyte population. **e** The fluorescence intensity corresponding to Ccl2 mRNA expression was increased in EAE-affected mice but was reverted to levels observed in naïve mice upon MSC treatment. Data are reported as means ± SD (n = 3, n = 4, n = 4; 3 sections each), and the Bonferroni post-test was used to compare all groups after two-way ANOVA (d) or one-way ANOVA (e). Clinical score of EAE-affected mice (mean cs 2.1) and MSC-treated mice (mean cs 2.0). TOPRO-3 nuclear counterstaining. Scale bar: a-c 20 µm

The dual RNAscope method enabled us to identify astrocytes at the protein level by GFAP immunostaining whilst assessing their expression of *Ccl2* at the mRNA level (Fig. 7a–c). The number of GFAP^−^/*Ccl2*^+^, GFAP^+^/*Ccl2*^+^, and GFAP^+^/*Ccl2*^−^ cells was calculated on randomly selected neocortex fields from naïve, EAE-affected, and EAE-affected MSC-treated mice (Fig. 7d). The results showed that MSC treatment was effective in counteracting the increase of both GFAP^−^/*Ccl2*^+^ microglia/monocyte/macrophage cells and GFAP^+^/*Ccl2*^+^ astrocytes in EAE-affected mice (Fig. 7d). The number of GFAP^+^/*Ccl2*^−^ astrocytes was increased in the neocortex of EAE-affected MSC-treated mice reaching the level detected in naïve mice (Fig. 7d). These data support our recent observations in vitro, that soluble factors produced by MSCs are able to reduce *Ccl2* expression in reactive astrocytes and in vivo, that treatment with MSCs can counteract the harmful prevalence of type A1 neurotoxic reactive astrocytes in the brain of EAE-affected mice [30]. In agreement with the morphometric results, the values of *Ccl2* expression, calculated as the mRNA probe’s fluorescence intensity, were similar in naïve and EAE-affected MSC-treated mice, whereas they were significantly higher in EAE-affected, untreated mice (Fig. 7e).

### Morphometric analysis of reactive CCL2^+^ microglia

The term microgliosis describes the morphological/functional and regionally dependent dynamic response of surveillant microglia which are able to change their morphology to hyper-ramified/hyper-complex (intermediate activated, hypertrophied, bushy) forms and/or to cells that completely lack processes (unramified, amoeboid) [62, 63]. The morpho-functional activation of microglial cells can be regionally dependent and also differs among cerebral cortex areas, where reactive, hypertrophic microglial cells may account for dysfunctional neurons [63].

**Fig. 8:**
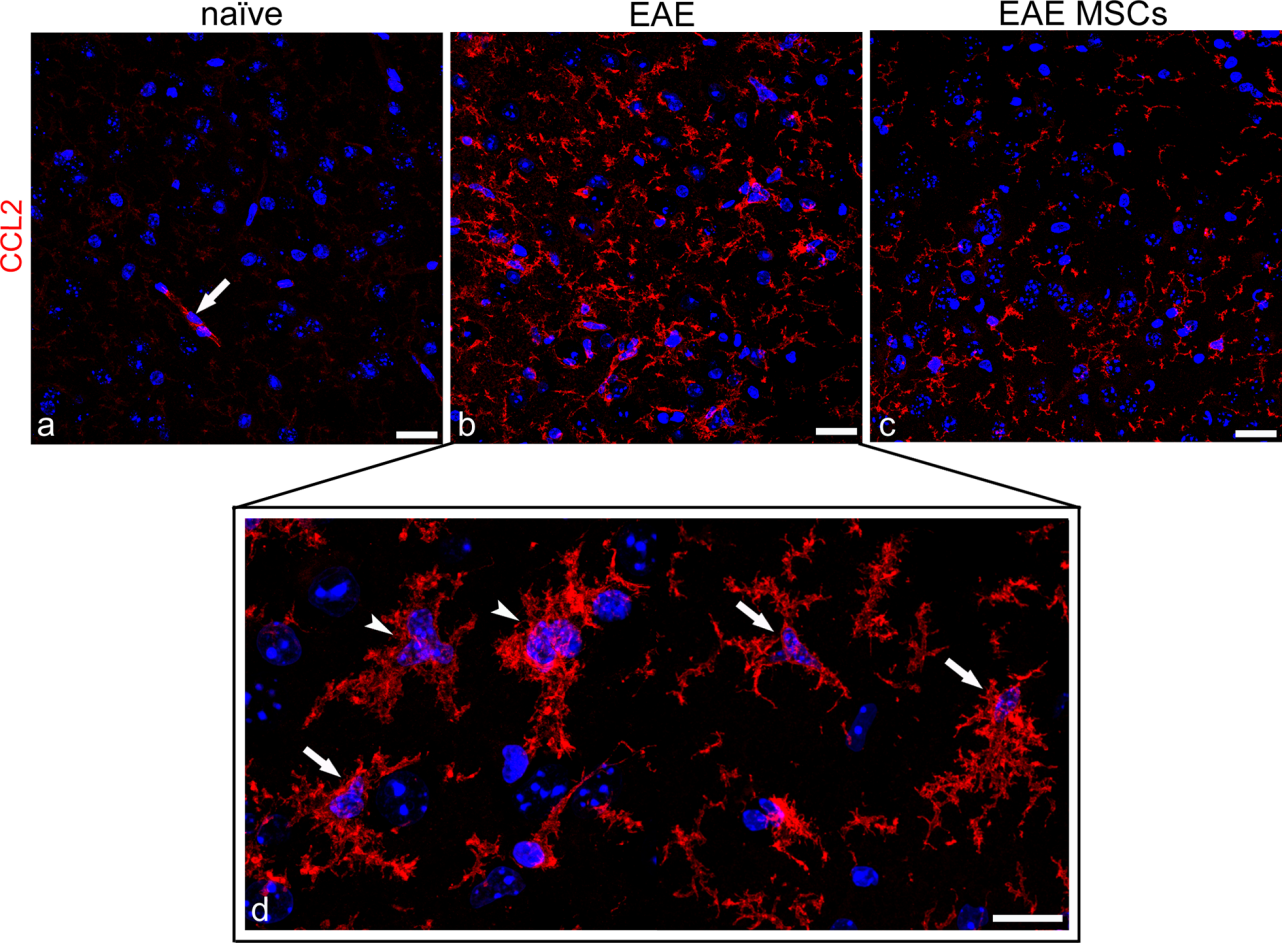
Representative images of CCL2 immunostained neocortex sections from naïve (a), EAE-affected (b, d; cs 3.5), and EAE-affected MSC-treated (c; cs 2.25) mice, sacrificed 24 h after MSC treatment. **a** A barely detectable CCL2 staining of surveillant microglia in the neocortex of naïve mice; note a CCL2-stained microvessel (*arrow*). **b**, **c** Strongly CCL2-stained, hypertrophic microglial cells dominate the neocortex of EAE-affected mice and, albeit with reduced features of microgliosis, are also clearly recognizable in the cortex of EAE-affected MSC-treated mice. **d** At a higher magnification, reactive microglial cells show hypertrophic profiles with stout, ‘thorny’ cell processes (*arrows*) and fuse to form cell clusters (*arrowheads*). TOPRO-3 nuclear counterstaining. Scale bars: a–c 25 µm; d 15 µm

Neocortex microglia morphology was investigated, searching for subtle morphological changes between MSC-treated and untreated EAE-affected mice, according to selected morphometric parameters, such as microglia convex hull area, circularity, number of endpoints, and immunofluorescence integrated intensity, on neocortex CCL2-stained sections from EAE-affected mice treated or not with MSC at 24 h from the disease onset (Fig. 8). The results showed that microglia activation was characterized by cell hypertrophy and a variety of morphological features, unveiled by the diffuse and strong expression of CCL2 (Fig. 8b, d). In EAE-affected mice, microglial cells reflected the state of neocortex neuroinflammation, acquiring relatively large soma and complex branched forms with tortuous, thicker primary processes endowed with wispy ends and thorny secondary and tertiary branches (Fig. 8d). MSC administration seemed to reduce microglia activation, reverting the cell morphology toward a less hypertrophic profile and downregulating CCL2 expression (Fig. 8c). The morphometric analysis showed that the mean microglia convex hull area (i.e., the polygonal area that includes a single microglial cell) was significantly reduced in MSC EAE (1149.02 ± 150.49 μm^2^) as compared with EAE-affected (3357.1 ± 569.28 μm^2^; *p* = 0.0003; n = 4) mice, indicating the beneficial effect of MSC treatment on microglia hypertrophy. The second chosen morphometric parameter, which denotes the cell circularity (i.e., the ratio between the area of the cell to the area of a circle having the same perimeter), was also significantly reduced in EAE MSC-treated (0.66 ± 0.02) compared with EAE-affected (0.77 ± 0.07; *p* = 0.031) mice, showing, in the latter, a morphology characterized by stout, thorny processes. This feature was also confirmed by the number of endpoints (number of process terminals/thorns *per* cell) that was significantly higher in EAE-affected (61.78 ± 26.08) than in EAE-affected MSC-treated (29.08 ± 1.62; *p* = 0.046) mice. These differences highlighted a state of neocortex microgliosis in EAE-affected mice, which diminished following MSC treatment. In addition to the morphological values, the extent of CCL2 staining, calculated as corrected total immunofluorescence density, showed high values of CCL2 in EAE-affected mice (550,079 ± 198,773 AU), which were stably decreased in EAE-affected MSC-treated mice (146,950 ± 44,333 AU; *p* = 0.0075); they correlated with the expression levels, obtained by the evaluation of *Ccl2* mRNA fluorescence intensity (see Fig. 7d). As suggested by the significantly diminished values obtained in EAE-affected mice, the MSC-based therapy attenuated the reactive profile of neocortex microglia, reducing the hyper-ramified/hyper-complex morphological aspect and the expression of CCL2.

### In-depth analysis of activated CCL2^+^ microglia interaction with the neocortex microvessels in EAE-affected mice treated or not with MSCs

**Fig. 9:**
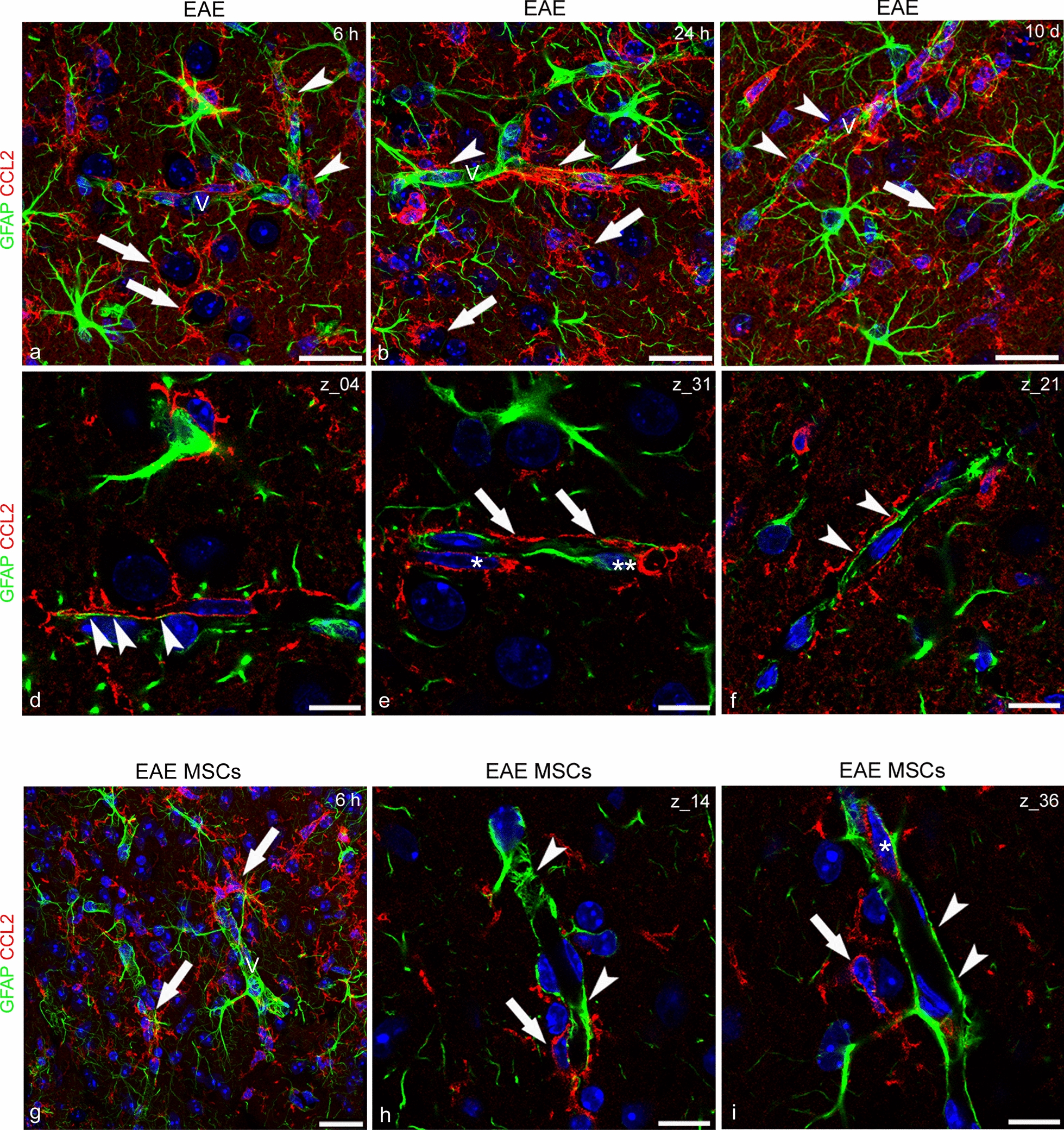
Representative images of neocortex sections, double immunostained for GFAP and CCL2, from EAE-affected mice (a-f; cs 1.5, 2.0, 2.5, respectively) sacrificed at 6 h (a, d), 24 h (b, e), and 10 days (c, f) from disease onset and EAE-affected MSC-treated (g-i; cs 1.5) mice, sacrificed at 6 h after MSC treatment. **a**–**c** Astrogliosis and microgliosis in EAE-affected mice involve cortex parenchyma and microvessels (V); perineuronal (*arrows*) and perivascular (*arrowheads*) microglia processes prevail in cortex fields. **d**-**f**, (details of a-c, respectively) Laser confocal single optical planes show alternate astrocyte (d, *arrowheads*) and microglial cell perivascular processes (e, *arrows*); there is an extensive vessel coverage by microglia and double-layered perivascular sheathes (f, *arrows*); note in (e) the perivascular microglia (*asterisk*) and astrocyte (*double asterisk*) bodies. **g** In EAE-affected MSC-treated mice, even though hypertrophic perivascular microglial cells are still recognizable (*arrows*), astrogliosis is reduced and perivascular astrocytes prevail on the vessel wall (V). **h**, **i** details of **g** Laser confocal single optical planes show a few perivascular microglial cells (*arrows*) close to a continuous astrocyte cover (*arrowheads*); note in (i) a CCL2-reactive endothelial cell (*asterisk*). TOPRO-3 nuclear counterstaining. Scale bars: a–c, g 25 µm; d–f, h–i 10 µm

Microglia, as one of the cell populations which contribute to define the neurovascular unit (NVU) of the CNS, mutually interact with all the other NVU cell components, primarily with astrocytes, with which they share a perivascular/juxtavascular position, abutting the parenchymal layer of the basal lamina, in both physiological and pathological conditions [64]. Interactions and reciprocal positions were revealed on CCL2/GFAP double stained cortex fields, analyzed on confocal projection images and, at higher resolution, on single optical planes from the z-axis image stacks. (Fig. 9). In the neocortex of EAE-affected mice, both perineuronal and perivascular astrocyte-microglia relationships reflected the severe condition of neocortex astrogliosis/microgliosis. Cortical neurons were ensheathed by microglia cells (Fig. 9a), and an overwhelming majority of perivascular CCL2^+^ microglial processes intruded within the NVU, doubled the perivascular astrocyte envelop (Fig. 9a–c), or directly contacted extensive tracts of the vessel wall (Fig. 9d–f). The main characteristic of the microglia perivascular sheath was ‘thorny’ processes that conferred a spiky appearance to the microvessel profile (Fig. 9a, f). This inflammatory scenario of widespread CCL2^+^ microglia was affected by MSC treatment, which seemed to minimize the presence of reactive microglia in the cortex parenchyma and at the microvessel interface, already at the earliest examined stage (6 h from disease onset/MSC injection) (Fig. 9g–i) and throughout the subsequent time intervals examined, 24 h and 10 days following MSC treatment, (data not shown). In-depth analysis of the neocortex microvessels in these mice showed that the outer CCL2^+^ microglial processes were almost completely lost, while astrocytes re-established their vascular domain, covering extensive vessel tracts and showing a reduced hypertrophic morphology (Fig. 9h, i). In accordance with these data, the tight association of perivascular astrocytes with the vessel wall, typical of the NVU in normal condition, was described as modified in MS patients, where most of the perivascular endfeet detach from the vessel wall and the vessel basal lamina is devoid of astrocyte endfeet [65].

### Assessment of structural and functional blood–brain barrier properties in MSC-treated and untreated EAE-affected mice

**Fig. 10:**
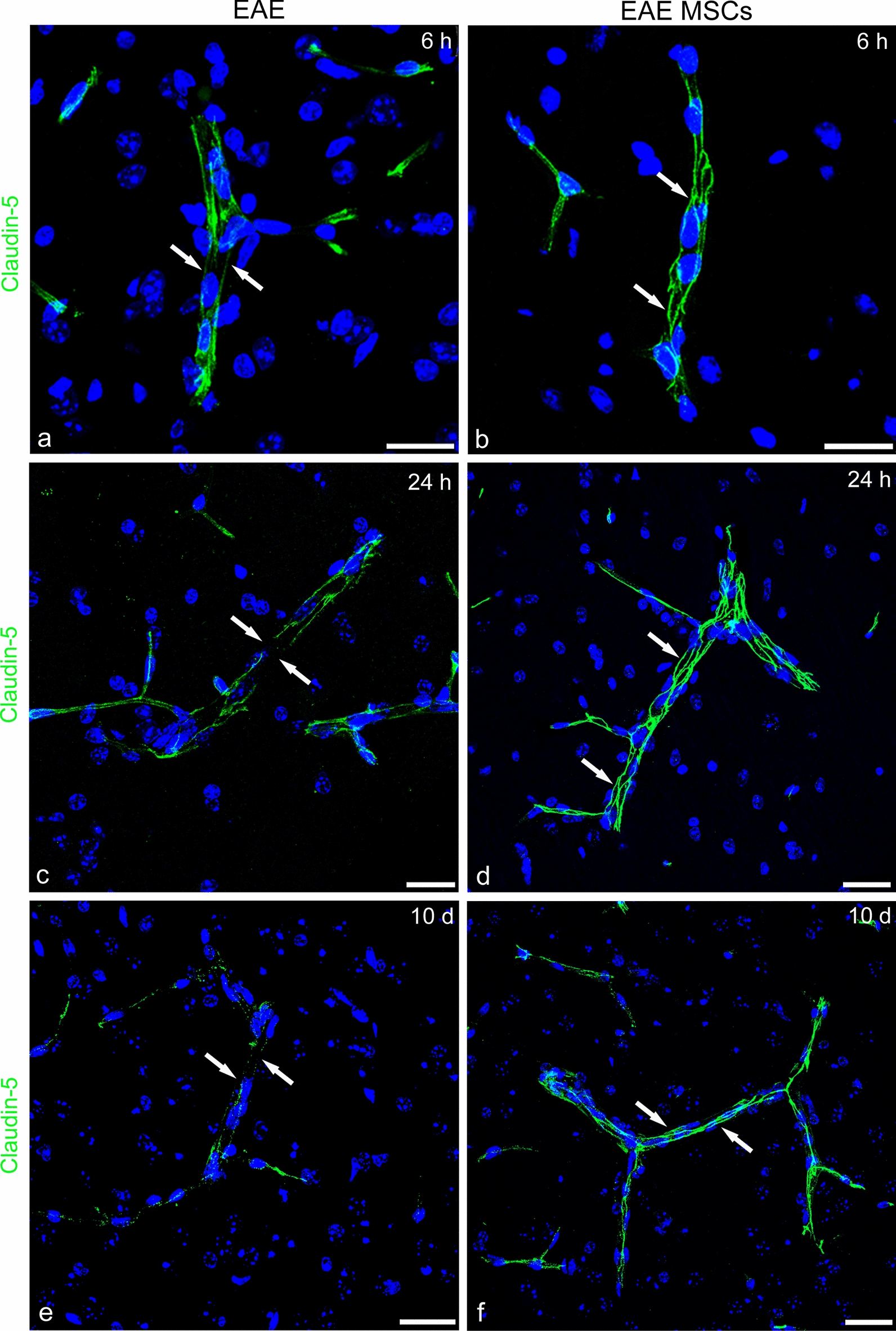
Representative images of claudin-5 immunostained neocortex sections from EAE-affected (a, c, e; cs 2.5, 3.5, 2.5, respectively) and EAE-affected MSC-treated (b, d, e; cs 2.0, 2.25, 2.25, respectively) mice, sacrificed at 6 h (6 h), 24 h (24 h), and 10 days (10d) after MSC treatment. **a**, **c**, **e** In EAE, the claudin-5 junctional staining pattern is lost at the shorter examined interval from the disease onset (a, *arrows*), as well as at the subsequent examined intervals (c, e; *arrows*). **b**, **d**, **f** A linear and continuous staining pattern for claudin-5 is recognizable on cortex microvessels of MSC-treated mice (*arrows*). TOPRO-3 nuclear counterstaining. Scale bars: a-f 25 µm

**Fig. 11:**
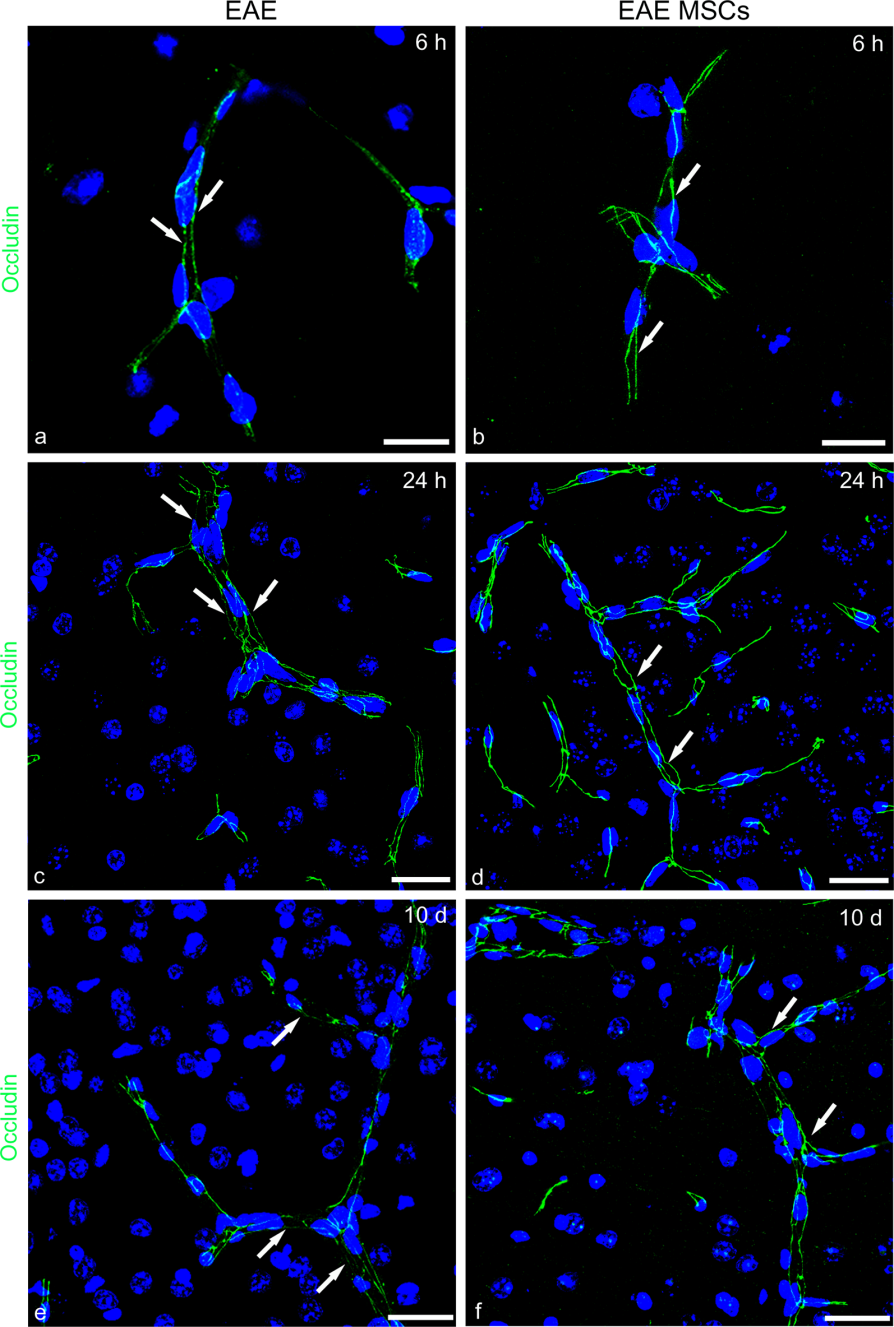
Representative images of neocortex sections from EAE-affected (a, c, e; cs 2.5,3.5, 2.5, respectively) and EAE-affected MSC-treated (b, d, e; cs 2.0, 2.25, 2.25, respectively) mice, sacrificed at 6 h (6 h), 24 h (24 h), and 10 days (10d) after MSC treatment, and immunostained for occludin. **a**, **c**, **e** In EAE-affected mice, the occludin junctional staining pattern is characterized by rows of fine puncta at 6 h and persists at 24 h and 10d (*arrows*). **b**, **d**, **f** Upon treatment with MSCs, the occludin pattern continuity is undisturbed (*arrows*). TOPRO-3 nuclear counterstaining. Scale bars: a–f 25 µm

**Fig. 12:**
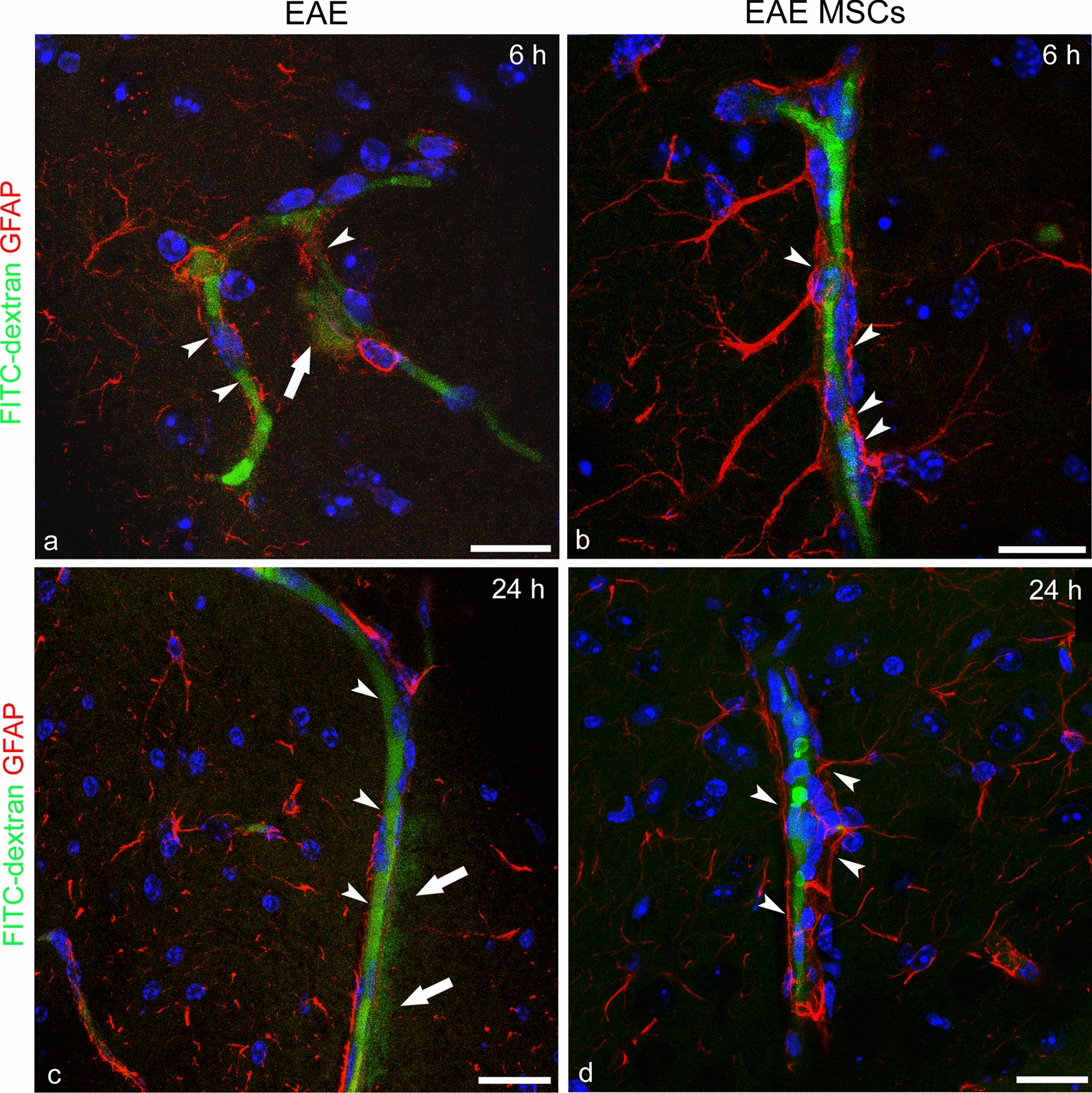
Representative images of neocortex sections from the FITC-dextran-injected experimental groups, immunolabelled for GFAP. (a, c; cs 1.5) EAE-affected and (b, d; cs 1.5) EAE-affected MSC-treated mice sacrificed at 6 h (6 h) and 24 h (24 h) after MSC treatment. **a**–**d** At both time points, the neocortex BBB-microvessels show traces of FITC-dextran leakage as a fluorescent halo in the surrounding parenchyma (a, c; *arrows*); in contrast, no trace of leakage and diffuse fluorescence is detectable in EAE-affected MSC-treated mice (b, d); note, in the latter, the preserved/restored continuity of the perivascular astrocyte endfeet (b, d; *arrowheads*) that is apparently lost in untreated EAE-affected mice (a, c; *arrowheads*). TOPRO-3 nuclear counterstaining. Scale bars: a–f 25 µm

BBB breakdown and dysfunction are well-known hallmarks of MS; their occurrence has been demonstrated to precede the formation of demyelinating lesions and involves white matter and adjacent gray matter as well as normal-appearing white matter [66, 67]. According to our previous studies carried out on the same model of MOG-induced EAE in mice, the BBB-microvessels of the neocortex show tight junction dismantling and increased permeability [11, 28, 29]. Recent studies have demonstrated that in the spinal cord of EAE-affected mice, MSC treatment ameliorates neuroinflammation, reduces demyelination, and protects BBB function, increasing TJ protein expression (31). In addition, CCL2, which has been suggested to work like a permeability-inducing molecule via its receptor CCR2 present on brain BBB endothelial cells [68, 69], may have a role in the BBB breakdown observed in the neocortex of EAE-affected mice. In the latter, both claudin-5 and occludin revealed discontinuous staining patterns (Figs. 10 and 11). Aspects of junction dismantling were observed at the earliest examined time point, 6 h from the disease onset, and were still detectable up to 10 days (Figs. 10 and 11a, c, e). MSC treatment possibly contributed to maintaining the linear and continuous junctional arrangement of claudin-5 and occludin strands (Figs. 10 and 11b, d, f). Accordingly, the permeability experiments with FITC-dextran and GFAP immunostaining in EAE-affected mice showed neocortex microvessels devoid of perivascular astrocyte endfeet and haloed by tracer extravasation (Fig. 12a, c). In EAE MSC-treated mice, FITC-dextran appeared restricted to the vessel lumen, and typical and robust astrocytes ensheathed the vascular wall (Fig. 12b, d).

## Discussion

### Microglia as the resident source of CCL2 in neocortex neuroinflammation

Several studies have focused on unveiling the cellular source(s) of CCL2 in inflammatory conditions, including in MS and EAE. In the intact CNS, in situ hybridization showed that in EAE, CCL2 is expressed by neurons and astrocytes [70, 71], thereby opening the view to mechanisms of endothelial transcytosis of the chemokine produced on the abluminal microvessel side [72, 73]. Advanced cell-source studies using two-cell- (astrocytes and endothelial cells) conditional CCL2 knockout mice later confirmed that both these cell types express the chemokine [74]. In addition, specific roles for CCL2 have been demonstrated in cell extravasation at diverse phases of inflammation, according to its endothelial or astrocyte origin [77]. However, how the differential activity of the cell-specific-derived CCL2 pools can be regulated and balanced is still unclear [74–77]. EAE-affected mice with a conditional genetic deletion of *Ccl2* in astrocytes have less macrophage and T-cell inflammation in the white matter of the spinal cord, less diffuse activation of astrocytes and microglia, and show a less severe disease at late stage, while having a similar incidence and severity of disease at onset, as compared to wild type EAE-affected controls [78]. However, a contribution by other resident cells and non-parenchymal cell types cannot be excluded, as global knockout mice for *Ccl2* and wild-type mice treated with anti-CCL2 antibodies display less severe experimentally induced neuroinflammatory diseases [79, 80].

It is not known whether cerebral cortex lesions in MS may involve the expression of CCL2, as cortex lesions have been not characterized in studies that described CCL2 expression (17), and CCL2 expression associated to microglia activation has not been investigated in studies that evaluated the levels of pro-inflammatory cytokines in different brain areas, including the cerebral cortex, in experimental conditions of systemic neuroinflammation [81]. In the present study, we have demonstrated that in the neocortex of EAE-affected mice, despite scarce leukocyte transmigration, neuroinflammation is characterized by a remarkable expression of CCL2 that spreads throughout the neocortex layers. Based on a battery of immunohistochemistry and in situ hybridization analyses, we show that the primary origin of CCL2 is the ‘real’ resident microglial cells that display an activated and reactive, non-amoeboid phenotype and express high levels of the chemokine. Microglia, as the resident CNS macrophages, play a critical role in neocortex neuroinflammation, responding to pathological noxae by prompt activation. However, the identification of resident microglia, although ontogenetically distinct from monocyte-derived macrophages, has always failed due to the absence of specific markers, regardless of their resting or activated state. In fact, activated microglia and ramified brain macrophages are nearly indistinguishable by both morphology and cell-surface markers, and even one of the most utilized microglia markers, the ionized calcium-binding adaptor molecule 1 (IBA1), does not allow differentiation between resident microglia and infiltrating blood-derived macrophages [82]. Recently, Satoh and colleagues [58], investigating the mouse microglia transcriptome datasets, identified *Tmem119* as a reliable microglia-specific marker, not expressed by other immune cells and detectable in all the microglia populations (resting, activated, amoeboid). In the same year, Buttgereit and colleagues [59] identified a microglial signature gene, *Sall1*, not expressed by the monocyte/macrophage system, restricted to microglia in the hematopoietic compartment and in the CNS, and critical in the control of the adult, resting microglia phenotype. We took advantage of these two reliable microglia markers, demonstrating that in the neocortex of EAE-affected mice, CCL2 immunostained cells co-expressed both markers, confirming these neocortex cells as ‘real’ resident microglial cells. Interestingly, the ‘shutdown’ effect of MSC-treatment on the expression of CCL2, reverted the reactive microglia from a TMEM119^+^/CCL2^+^ to a TMEM119^+^/CCL2¯ phenotype, a milder inflammatory condition, albeit with signs of cell hypertrophy. The results obtained with SALL1, whose expression may score the neocortex neuroinflammatory state, were consistent with these data. The expression of SALL1 has been already investigated in EAE neuroinflammation: in this context, microglia maintained the *Sall1* transcriptional signature, which did not appear to be induced by the CNS microenvironment, and was distinct from that of CNS-invading myeloid cells, including recruited neutrophils and monocyte-derived cells, as well as from that of microglia-like cells after microglia deprivation [59]. Our analysis explored the SALL1 and IBA1 populations of cells, demonstrating that the population mainly present in the neocortex of EAE-affected mice was SALL1^+^/IBA1^+^, the same population found to be effectively reduced by MSC-treatment, whereas the percentage of SALL1^−^/IBA1^+^ monocyte/macrophage cells did not change in MSC-treated and not treated mice. According to the results obtained by fluorescence intensity measurement, SALL1 immunostaining was found to be reduced in EAE-affected mice, whereas it was even significantly higher in EAE-affected MSC-treated mice than in naïve mice. Interestingly, immunohistochemistry of brain sections from *Sall1*^GFP/+^ mice has shown that, during embryogenesis, neuronal and glial progenitors evince a high expression of *Sall1* [83]. These data, together with the reported role of SALL1 as a transcriptional regulator of the non-reactive state of microglia able to silence inflammatory programs in CNS [59], suggest that MSC-treatment is essential in the transition from activated to homeostatic microglia and, further, to a protective, developmental microglia subset [84].

In this study, in parallel with the identification of microglia as the source of CCL2, colocalization experiments and IHC/ISH analyses suggested that other cells express CCL2, albeit at minimal levels. As expected, CCL2 was colocalized with monocyte/macrophage/microglia markers on the venular, perivascular infiltrates, observed at the border between the deepest cortex layers and the subcortical white matter in EAE-affected treated and non-treated mice, and with endothelial CD31 on neocortex microvessels of naïve mice. Moreover, dual RNAscope analysis carried out at protein (GFAP) and mRNA (*Ccl2*) levels, revealed a subpopulation of GFAP^+^/*Ccl2*^+^ in EAE-affected treated and non-treated mice, without differences between the two experimental groups, while in EAE-affected MSC-treated mice the population of GFAP^+^/*Ccl2*^−^, which was minimal in EAE-affected mice, rose to the same levels observed in naïve controls. It is conceivable that MSCs may impact the critical balance between neurotoxic A1 astrocytes and protective A2 astrocytes. These two opposite phenotypes may be included in the detected GFAP^+^/*Ccl2*^+^ (A1) and the GFAP^+^/*Ccl2*^*−*^ (A2) subsets of astrocytes, with the latter being increased in EAE-affected MSC-treated mice and, therefore, possibly involved in diminishing the level of neocortex inflammation.

### Microglia activation, intermediate and reactive states, in neocortex microgliosis

Microglia are macrophage-like resident immune cells that not only work as antigen-presenting cells but are also effector cells in neurological disorders. The accumulation of activated microglia (microgliosis) is a major pathological hallmark of neurodegenerative diseases [85–87]. In support of the concept of highly dynamic surveillant microglia that continuously explore their surroundings in the homeostatic state, activated (reactive) microglia assume multiple forms in response to virtually any kind of CNS pathology. In vivo, microglial activation causes cell hyper-ramification (intermediate stage) and/or a reactive, hypertrophic phenotype, with cell body swelling, thickening of proximal processes, a reduction in distal ramification, the formation of bipolar/tripolar microglial rod cells, and cell clustering [62, 63]. Eventually, microglia may transform morphologically into large amoeboid forms that work as brain macrophages. This latter ‘classically activated’ microglia type is thought to be critical for the phagocytosis of myelin, antigen presentation to T cells, and the release of proinflammatory cytokines in active lesions [88–93]. With regard to more subtle morphological features of microglia in their intermediate/hypertrophic state of activation, these have been quantitated applying multifractal analyses to assess the forms that these cells can adopt by extending and retracting fine and gross processes, in relation to their variety of roles in both physiological and pathological conditions [62]. Thus, ‘spider-like’, ramified surveillant and the ‘amorphous blobs’, de-ramified reactive/amoeboid microglia are simply the opposite poles of a variety of complex morphologies, dynamically reversible at any point, depending on changes in the pathological condition/location [62]. In its reactive hypertrophic and non-amoeboid form, microglia release cytokines/chemokines, including CCL2, CCL5, nucleic acids, excitatory amino acids, reactive oxygen species, TNF-α, transforming growth factor β (TGF-β), interferon γ, IL-1α, -5, -6, -10, -12, -18, macrophage inflammatory protein 1α, metalloproteases, nitric oxide, and peroxynitrite [94]. It has been demonstrated that among these secreted factors, IL-1α, TNF-α, and complement component 1, subcomponent q (C1q) are all required as initiators of neurotoxic A1 astrocytes induction in vivo*,* and that A1 astrocytes significantly decrease in number only when treated with the anti-inflammatory TGF-β or fibroblast growth factor (FGF) [95]. In light of this evidence, it can be hypothesized that the effect of MSCs is mediated by a possibly still unknown inducer of the protective A2 phenotype (see also [30].

### Neocortex microgliosis and neuronal and BBB dysfunction

In MOG-induced EAE, CCL2-expressing microglia surround the cortex neurons as a potential sign that glutamatergic neurons are becoming dysfunctional [96]. In fact, as demonstrated in a variety of neurological disorders, including neurodegenerative diseases, characterized by a kind of neurotoxicity called ‘excito-neurotoxicity’, activated hypertrophic microglia accumulate around neurons and, by releasing glutamate, impair the cortex glutamatergic pathways, thus contributing to the pathogenesis of cognitive deficits, schizophrenia, and bipolar disorders [94]. It is worth mentioning that in the ‘microglia hypothesis’ of schizophrenia, the disease occurs in MS patients with a prevalence (> 60%) of associated frontotemporal lesions [97, 98], a pathogenesis supported by the demonstrated CCL2-driven microgliosis observed in the neocortex of EAE-affected mice.

Microvascular microgliosis seems to show the same ‘invasiveness’ as microglia spreading among the cortex neurons. Thus, in EAE-affected mice, at the microvessel level, hypertrophic microglia always recognizable by a high CCL2 expression surrounded the microvessel wall to form an almost continuous envelope competing with perivascular astrocytes at the glia *limitans* front. CCL2 released by neocortex perivascular and juxtavascular [intermediate forms of perivascular microglia that acquire an elongated conformation, distinguishing them from perivascular ramified microglia [62]] microglia subpopulations, may act in an autocrine as well as a paracrine manner, potentially involving, through the CCR2 receptor, all the cellular components of the NVU, including endothelial cells and pericytes, perivascular astrocytes, OPCs, and macrophages. It has been demonstrated in in vitro models of human BBB and HIV infection that endothelial permeability increases not simply upon addition of CCL2, but rather through the presence of a CCL2 gradient, which correlates with BBB disruption, as evidenced by modified tight junction proteins and enhanced vessel permeability [99].

The prevalence of a microglial CCL2 gradient in the context of the observed microvascular microgliosis could be related to the levels of expression of CCL2 by endothelial cells. In agreement with our observations, under normal conditions, endothelial cells constitutively express well detectable levels of CCL2 [100]; however, during neuroinflammation, the endothelial expression of CCL2 seemed to be downregulated, whereas there was an overwhelming presence of microglia-derived CCL2 in the perivascular microenvironment. Using cultured brain microvascular endothelial cells, it has been observed that exogenously added CCL2 depressed the release of endogenous CCL2, and further caused diminished C*cl2* mRNA levels in endothelial cells [101]. It is difficult to explain the reason for the observed baseline level of CCL2 in endothelial cells under physiological conditions, although it could serve to neutralize CCR2 receptors present on immune cells, thereby preventing their entry into the CNS. During EAE, a similar ‘paradoxal’ effect may result from the overwhelming presence of CCL2 in the neocortex microvessel surroundings, together with the enhanced endothelial cell uptake of CCL2 from the abluminal to the luminal vessel side [101, 102]. The combined effects of perivascular release of CCL2 by microglia and abluminal-luminal endothelial transport of the chemokine may create a concentration gradient between the neocortex parenchyma and the bloodstream; this would increase the efflux of CCL2 at the luminal endothelial surface and the levels of the chemokine in the blood, since constitutive CCL2 is cleared from the blood through uptake by the endothelial cells in a CCR2-dependent manner [103]. Although CCL2 secretion is counteracted by a constant uptake and internalization by CCR2-expressing cells, the effectiveness of CCL2 clearance in disease settings depends upon this critical equilibrium (endothelial cells are inefficient in clearing excess CCL2 from the blood at rates equal to the CCL2 efflux), which may easily be impaired by the surplus. This may determine an imbalance between the secretion of CCL2 and its CCR2-mediated uptake, and hence an increase in CCL2 blood levels and the spread of the pro-inflammatory chemokine in all the areas of the CNS [103]. Moreover, in areas where CCL2 is produced in absence of an inflammatory infiltrate, this dissociation may be due to internalization and desensitization of endothelial CCR2 by transcytosed CCL2, a condition that has been suggested to impair transendothelial migration [73].

The disruption of BBB observed in the neocortex of EAE-affected mice, and the prompt barrier recovery documented in EAE-affected MSC-treated mice, can also be mediated by microglia-derived CCL2 and by the treatment-related control of microgliosis, respectively. It is well known that CCL2 can mediate BBB disruption in a variety of pathological conditions [69, 104], including through subcellular redistribution of tight junction and tight junction-associated proteins and involvement of RhoA/protein kinase C-α pathway [105]. As already demonstrated for pericyte- and astrocyte-derived CCL2 in multiple cultures with brain endothelial cells, where CCL2 was primarily luminal [100], microglia-derived CCL2 could be present at high levels in the luminal compartment, following the same mechanisms unveiled for the astrocyte-pericyte cross-talk and endothelial cell transport. An interesting speculation is whether, and to what extent, the paracrine effect of MSCs may preserve BBB integrity and reduce neuroinflammation and CCL2-driven microgliosis by means of secreting neuroprotective molecules [106].

### Microglia response to treatment of EAE with MSCs

Systemic administration of bone marrow-derived MSCs has proven its therapeutic efficacy in MS pre-clinical models [33, 107, 108] and is a non-invasive method for treatment of diseases affecting organs such as the brain. Delineating regionally dependent microglia subtypes could be of particular importance to gaining a further understanding of the heterogeneous response and the differential contribution of cell subsets to the disease. This concept is supported by our results in MOG-induced EAE, where microglia rather than astrocytes played a prime role in sustaining neuroinflammation and possibly neuronal damage and BBB impairment. After treatment with MSCs, the massive microgliosis observed in the neocortex of EAE-affected mice appears to be effectively counteracted, featuring reduced cell hypertrophy, reduced IBA1 and *Ccl2*/CCL2 expression, increased neuroprotective SALL1, and reduced NVU adjoining processes. Thus, microglia respond directly to MSC treatment, or indirectly, through resident cell effectors such as astrocytes that have been recently demonstrated to respond to MSC treatment, reacquiring stem cell properties and protective and reparative actions [30]. Direct modulation of the microglia phenotype by MSCs has been demonstrated in vitro in co-cultures of microglia and MSCs [41]. In this condition, a direct MSC-microglia paracrine crosstalk impairs microglia activation and inhibits the expression and release of inflammatory molecules, switching microglia from a detrimental to a neuroprotective phenotype.

## Conclusions

One of the most significant findings in this study was the clear association between a condition of neocortex microgliosis, as the response to neuroinflammatory cues, and the role of the intermediate/hypertrophic and CCL2-expressing microglia in affecting neuronal and microvascular components during EAE. The elegant study by Nimmerjahn and colleagues [93] has shown, using in vivo two-photon imaging of the neocortex, that microglial cells are promptly activated even in their presumed resting state, continually changing the position, number, and shape of their processes, and that BBB disruption provokes an immediate activation of microglia, not necessarily evoked by a change to an amoeboid morphology. It has also been postulated that the mere presence of a chemokine does not necessarily mean that it is available to receptors at any site to promote lymphocyte recruitment; Th2 cells are not equally well recruited to the CNS by their population-selective chemokines, CCL2, CCL7, and CCL8 [109]. To fully understand the role of microglia and microglia-derived CCL2 in EAE and MS, it is necessary to distinguish immune cell recruitment functions from other key effects on the neuronal and vascular neocortex components. A better understanding of these complex activities may suggest therapeutic applications across a wide spectrum of neurodegenerative diseases that affect neocortex homeostasis, connectivity, and function.

## Technical limitations

In our investigation, we used a powerful approach, namely the dual IHC/ISH method based on RNAscope technology, adapted to thick free-floating brain sections. (46), which we partly modified (see Methods chapter). However, the use of specific cell markers (i.e., TMEM119 and SALL1) was impaired by the sensitivity of their epitopes to the RNAscope protocol. This resulted in the disappearance of the cell-specific staining, thereby reducing the efficacy of the concomitant detection of *Ccl2*. Therefore, the demonstration of CCL2 expression focused on astrocytes detected by GFAP and the demonstration of *Ccl2* mRNA in microglial cells was indirect.

## Supplementary Information

**Additional file 1: Figure S1.** Representative confocal microscopy images of immunolocalization of MSCs in the lung of EAE-affected MSC-treated mice (cs 1.5, 2.25). MSCs were retrovirally transduced to permanently express green fluorescent protein (GFP) and delivered intravenously to mice. At 24 h from disease onset/MSC treatment, a low GFP inherent fluorescence is detectable (a, *arrow*s), whereas IHC with an anti-GFP antibody (b, c) shows distinct rounded cell profiles, with a large cytoplasm/nucleus ratio and a GFP-positive, fluorescent granular pattern (macrophage autofluorescence was below the photography threshold) in the lung septa. TOPRO-3 nuclear counterstaining. Scale bars: a, b 20 µm; c 10 µm.

**Additional file 2: Figure S2.** Representative images of neocortex sections from naïve (a, d, g), EAE-affected (b, e, h; cs 1.5, 2.0, 2.5, respectively), and EAE-affected MSC-treated (c, f, i; cs 1.0, 1.50, 2.25, respectively) mice, sacrificed at 6 h (a-c), 24 h (d-f), and 10 days (g-i) after MSC treatment, double immunostained for collagen type IV (COL IV) and CCL2. (a, d, g) In naïve mice, the endothelial cells of neocortex microvessels (V) show a constitutive expression of CCL2. (b, e, h) In EAE-affected mice, only a few of the endothelial cells along the vessel length are stained by CCL2; instead, the chemokine appears to be expressed by hypertrophic, highly ramified cells scattered in the neocortex parenchyma and located in a perivascular position (*arrows*). (c, f, i) In EAE-affected MSC-treated mice, the neocortex microvessel (V) are also primarily CCL2-negative, while a lower staining for the chemokine and reduced signs of hypertrophy characterizes microglia-like cells (*arrows*). TOPRO-3 nuclear counterstaining. Scale bars: a-i 25 µm.

**Additional file 3: Figure S3.** Representative images of neocortex sections from naïve mice (a), EAE-affected (b; cs 3.5) and EAE-affected MSC-treated (c; cs 3.0) mice, sacrificed at 24 h after MSC treatment and double immunostained for CD31 and CCL2. (a) CD31^+^ endothelial cells show a constitutive expression of CCL2 in naïve mice. (b, c) In EAE-affected mice treated or not with MSCs, most of the CD31-stained endothelial cells are CCL2^−^, while CCL2-stained microglia-like cells surround the vessel wall. TOPRO-3 nuclear counterstaining. Scale bars: 25 µm.

## Data Availability

The datasets used and/or analysed during the current study are available from corresponding author on reasonable request.

## Abbreviations

BBB: Blood–brain barrier
CCL2: C–C motif chemokine 2
CCR2: C–C motif chemokine receptor type 2
CNS: Central nervous system
EAE: Experimental autoimmune encephalomyelitis
GFAP: Glial fibrillary acidic protein
IBA1: Ionized calcium binding adapter molecule 1
IHC: Immunohistochemistry
ISH: In situ hybridization
MOG: Myelin oligodendrocyte glycoprotein
MS: Multiple sclerosis
MSCs: Mesenchymal stem cells
NG-2: Neuron-glial antigen 2
NVU: Neurovascular unit
OPCs: Oligodendrocyte precursor cells
SALL-1: Sal-like protein 1
TMEM119: Transmembrane protein 119
TJs: Tight junctions
