# Supplementary figures and images for: Microglia-derived CCL2 has a prime role in neocortex neuroinflammation
Source: Fluids Barriers CNS. 2022 Aug 30;19:68. doi: 10.1186/s12987-022-00365-5 (PMC9429625; doi:10.1186/s12987-022-00365-5)

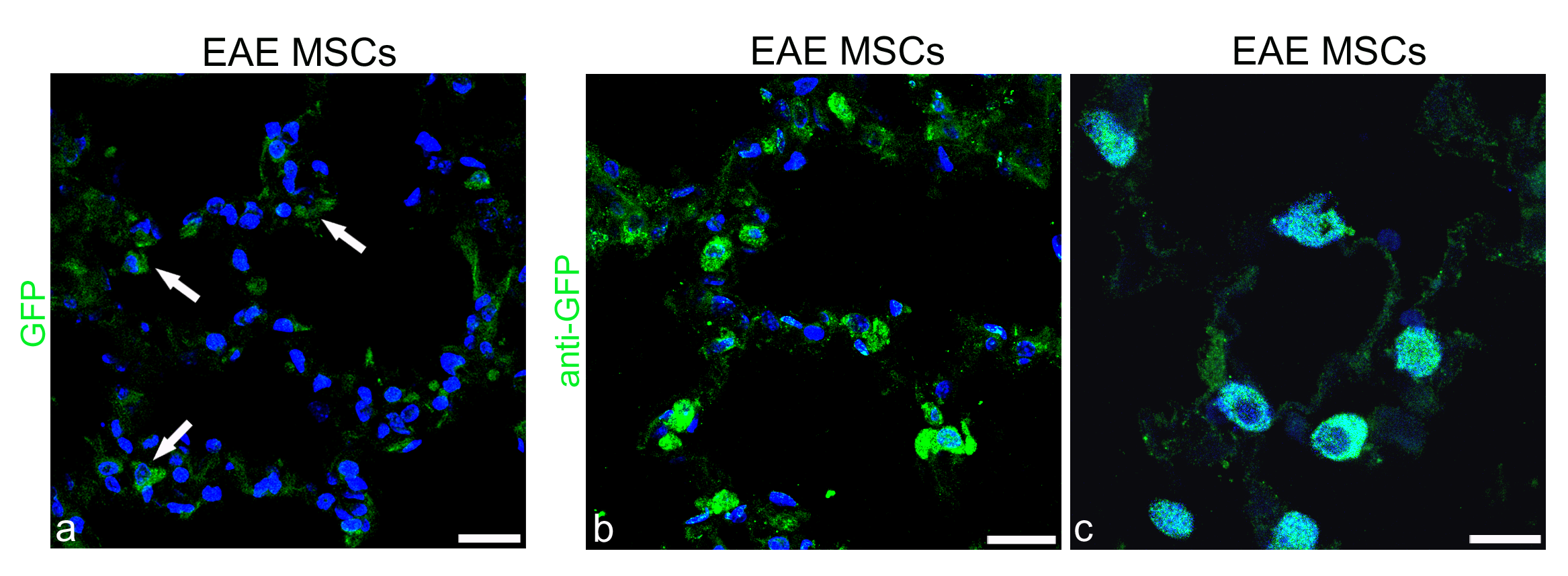

Supplement: Supplementary file 1 — Additional file 1: Figure S1. Representative confocal microscopy images of immunolocalization of MSCs in the lung of EAE-affected MSC-treated mice (cs 1.5, 2.25). MSCs were retrovirally transduced to permanently express green fluorescent protein (GFP) and delivered intravenously to mice. At 24 h from disease onset/MSC treatment, a low GFP inherent fluorescence is detectable (a, arrows), whereas IHC with an anti-GFP antibody (b, c) shows distinct rounded cell profiles, with a large cytoplasm/nucleus ratio and a GFP-positive, fluorescent granular pattern (macrophage autofluorescence was below the photography threshold) in the lung septa. TOPRO-3 nuclear counterstaining. Scale bars: a, b 20 µm; c 10 µm. [file 12987_2022_365_MOESM1_ESM.tif]

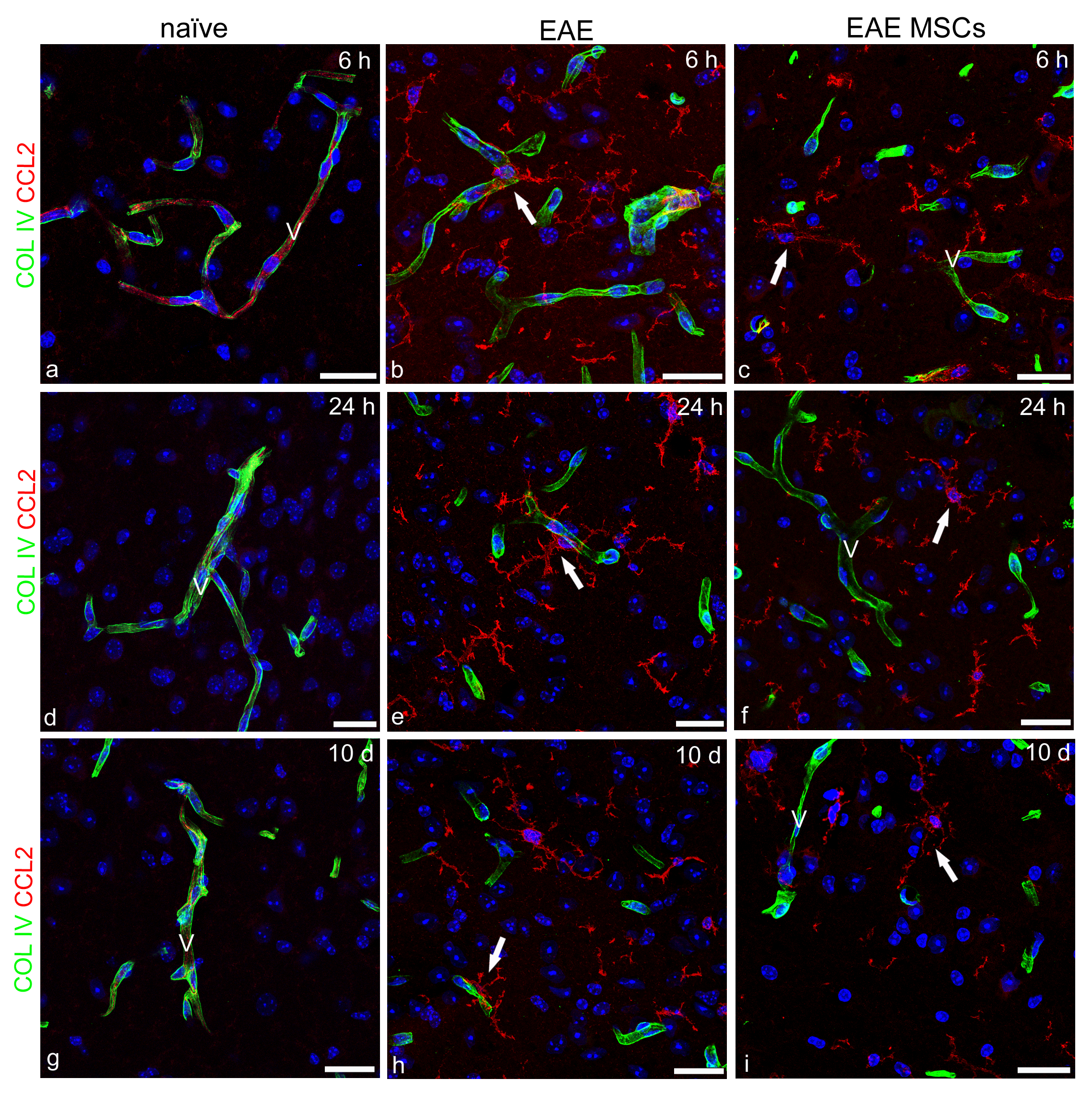

Supplement: Supplementary file 2 — Additional file 2: Figure S2. Representative images of neocortex sections from naïve (a, d, g), EAE-affected (b, e, h; cs 1.5, 2.0, 2.5, respectively), and EAE-affected MSC-treated (c, f, i; cs 1.0, 1.50, 2.25, respectively) mice, sacrificed at 6 h (a-c), 24 h (d-f), and 10 days (g-i) after MSC treatment, double immunostained for collagen type IV (COL IV) and CCL2. (a, d, g) In naïve mice, the endothelial cells of neocortex microvessels (V) show a constitutive expression of CCL2. (b, e, h) In EAE-affected mice, only a few of the endothelial cells along the vessel length are stained by CCL2; instead, the chemokine appears to be expressed by hypertrophic, highly ramified cells scattered in the neocortex parenchyma and located in a perivascular position (arrows). (c, f, i) In EAE-affected MSC-treated mice, the neocortex microvessel (V) are also primarily CCL2-negative, while a lower staining for the chemokine and reduced signs of hypertrophy characterizes microglia-like cells (arrows). TOPRO-3 nuclear counterstaining. Scale bars: a-i 25 µm. [file 12987_2022_365_MOESM2_ESM.tif]

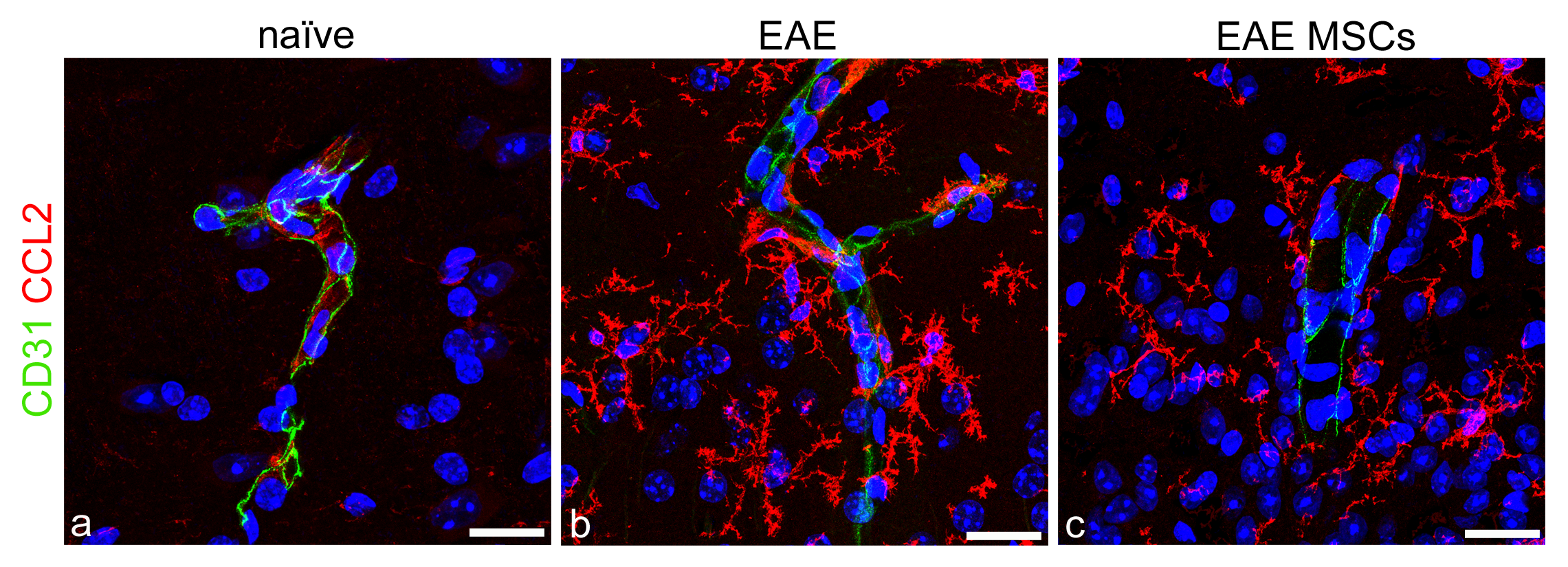

Supplement: Supplementary file 3 — Additional file 3: Figure S3. Representative images of neocortex sections from naïve mice (a), EAE-affected (b; cs 3.5) and EAE-affected MSC-treated (c; cs 3.0) mice, sacrificed at 24 h after MSC treatment and double immunostained for CD31 and CCL2. (a) CD31+ endothelial cells show a constitutive expression of CCL2 in naïve mice. (b, c) In EAE-affected mice treated or not with MSCs, most of the CD31-stained endothelial cells are CCL2−, while CCL2-stained microglia-like cells surround the vessel wall. TOPRO-3 nuclear counterstaining. Scale bars: 25 µm. [file 12987_2022_365_MOESM3_ESM.tif]
